# Bisarylureas Based on 1*H-*Pyrazolo[3,4-*d*]pyrimidine Scaffold as Novel Pan-RAF Inhibitors with Potent Anti-Proliferative Activities: Structure-Based Design, Synthesis, Biological Evaluation and Molecular Modelling Studies

**DOI:** 10.3390/molecules22040542

**Published:** 2017-03-29

**Authors:** Yu Fu, Yuanyuan Wang, Shanhe Wan, Zhonghuang Li, Guangfa Wang, Jiajie Zhang, Xiaoyun Wu

**Affiliations:** Guangdong Provincial Key Laboratory of New Drug Screening, School of Pharmaceutical Science, Southern Medical University, Guangzhou 510515, China; fuyuky0312@163.com (Y.F.); 15626047153@163.com (Y.W.); wansh@fimmu.com (S.W.); lzhuang@fimmu.com (Z.L.); wguangfa@smu.edu.cn (G.W.)

**Keywords:** DFG-out, pan-RAF inhibitor, 1*H*-pyrazolo[3,4-*d*]pyrimidine derivatives, biological activities, molecular docking, molecular dynamics simulation

## Abstract

RAF (Ras activating factor) kinases are important and attractive targets for cancer therapy. With the aim of discovering RAF inhibitors that bind to DFG-out inactive conformation created by the movement of Asp-Phe-Gly (DFG), we conducted structure-based drug design using the X-ray cocrystal structures of BRAF (v-raf murine sarcoma viral oncogene homolog B1), starting from bisarylurea derivative based on 1*H*-pyrazolo[3,4-*d*]pyrimidine scaffold **1a**. Most of the synthesized compounds showed good to excellent inhibitory activities against BRAF^V600E^ kinase, possessed moderate to potent anti-proliferative activities against four tumor cell lines (A375, HT-29, PC-3 and A549) and good selectivity towards cancer cells rather normal cells (Madin-Darby canine kidney, MDCK). The most promising compound, **1v**, exhibited potent inhibitory activity against not only BRAF^V600E^ (half maximal inhibitory concentration, IC_50_ = 23.6 nM) but also wild-type BRAF (IC_50_ = 51.5 nM) and C-RAF (IC_50_ = 8.5 nM), and effective cellular anti-proliferative activities against A375, HT-29, PC-3 and A549 cell lines as well as a very good selectivity profile. Moreover, compound **1v** mainly arrested the A375 cell line in the G0/G1 stage, and showed significant suppression of MEK (mitogen-activated protein kinase kinase) phosphorylation in A375 and HT-29 cell lines. Taken together, the optimal compound **1v** showed excellent in vitro potency as a pan-RAF inhibitor. In addition, the promise of compound **1v** was further confirmed by molecular dynamics simulation and binding free energy calculations.

## 1. Introduction

The mitogen-activated protein kinase (MAPK) cascades consist of Ras/RAF(Ras activating factor)/MEK(mitogen-activated protein kinase kinase)/ERK(extracellular receptor kinase) signal transduction and play an important role in the regulation of cellular activities, including proliferation, differentiation, and survival [1,2]. There are three RAF isoforms, ARAF, BRAF (v-raf murine sarcoma viral oncogene homolog B1), and CRAF (RAF-1), which play central roles in this cascade [3,4]. Among them, BRAF isoform is more easily activated by Ras and has higher basal kinase activity than the other two isoforms, which provides a reasonable explanation for the mutational activation of BRAF frequently observed in human tumors. The BRAF genetic mutation is present in about seven percent of all human cancers, and the most common mutation BRAF^V600E^ is involved in various carcinomas, including metastatic melanoma, thyroid cancer, and colon cancer [5]. This V600E point mutation results in constitutive kinase activity 500-fold greater than wild type BRAF [6], which also closely correlates with the increase of malignancy and the decrease of response to chemotherapy [7,8]. Hence, targeting Ras/RAF/MEK/ERK signal transduction may be a legitimate approach to cancer treatment.

**Figure 1:**
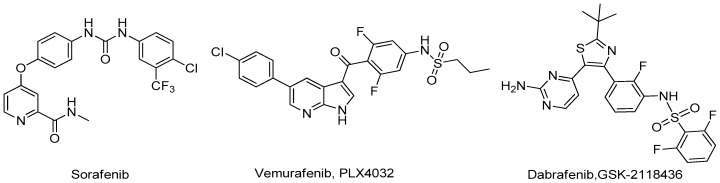
BRAF inhibitors.

In the last decade, many small-molecule inhibitors targeting RAF have been developed. Sorafenib (Figure 1) [9], as the first RAF kinase inhibitor, was approved by the Food and Drug Administration (FDA) for the treatment of advanced renal cell carcinoma (RCC) in 2005, unresectable hepatocellular carcinomas (HCC) in 2007 and thyroid cancer [10]. Sorafenib is classified as a type II inhibitor [11,12], which binds exclusively an inactive DFG-out conformation created by the movement of Asp-Phe-Gly (DFG) and stabilizes its conformation. It not only binds at the adenosine triphosphate (ATP) binding site but also extends to the hydrophobic “back pocket” of protein created by the flip of the DFG motif. In contrast, vemurafenib [13,14,15] (PLX4032, Figure 1) and dabrafenib [16] (GSK-2118436, Figure 1) are classified as type I inhibitors, which bind to the DFG-in active conformation of the ATP binding site. However, these type I inhibitors are highly BRAF-selective. Vemurafenib and dabrafenib have been reported to cause rapid development of squamous cell carcinoma (SCC) and keratoacanthoma, which may be caused by paradoxical activation of the MAPK pathway by a selective inhibitor in cells bearing wild-type (WT) BRAF [17,18,19]. This feedback activation was significantly suppressed by DFG-out type pan-RAF inhibitors, but not by DFG-in type inhibitors. Therefore, we aimed at discovering DFG-out-type pan-RAF inhibitors.

**Figure 2:**
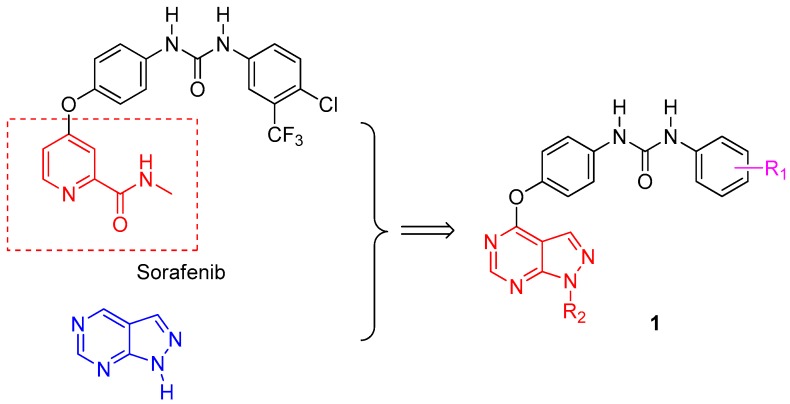
The design of target compounds.

**Figure 3:**
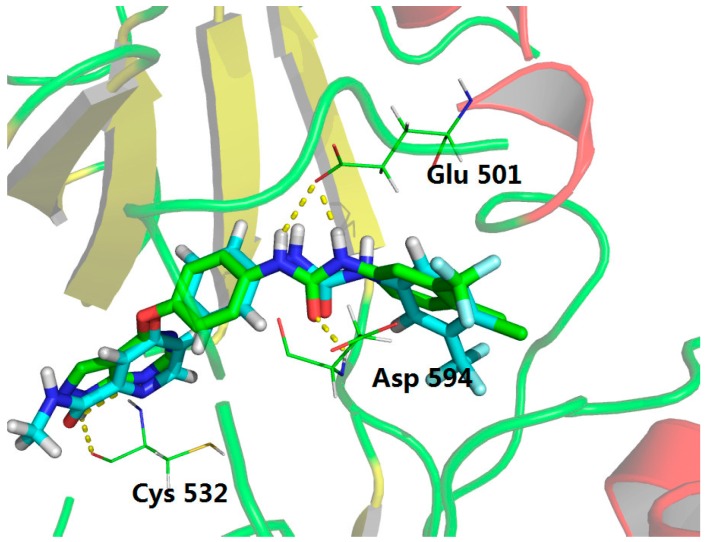
Overlap of the docked conformation of **1a** (green) and crystal structure of Sorafenib (cyan) bound to BRAF^V600E^ (Protein Data Bank (PDB) code: 1UWJ). Yellow dots represent hydrogen bonds. (BRAF^V600E^ in the colored cartoon, with ligands in the stick model, and the key residues in the line model).

The 1*H*-pyrazolo[3,4-*d*]pyrimidine nucleus is an important drug-like scaffold present in many pharmacologically active compounds, and the antitumor activity was initially reported many years ago [20]. Having similar structure of the purines, 1*H*-pyrazolo[3,4-*d*]pyrimidine, a template of tyrosine kinase hinge-binding, was extensively chemically modified to improve the biological activity and kinase selectivity. To find potent RAF inhibitors, encouraged by the success of Sorafenib, we designed Sorafenib analogue **1a** by replacing the pyridine ring of Sorafenib with 1*H*-pyrazolo[3,4-*d*]pyrimidine moiety (Figure 2). To the best of our knowledge, although the inhibitory activities of **1a** and its analogues targeting VEGFR-2 (vascular endothelial growth factor receptor 2) and FLT3 (fms-related tyrosine kinase 3) have been reported [21], the RAF inhibitory activities of these derivatives are being reported for the first time by our group. Based on the promising activities as VEGFR-2 and FLT3 inhibitors with potent in vitro and in vivo activity against acute myeloid leukemia, we would like to examine the RAF inhibitory activities further. Model of **1a** in the active site of BRAF^V600E^ (Protein Data Bank (PDB) code: 1UWJ), which was reported to be in the DFG-out inactive conformation, was constructed by Surflex–Dock according to our previous protocol [22]. As shown in Figure 3, the model suggested that **1a** overlapped well with the co-crystal ligand sorafenib in the active site of BRAF^V600E^. The 1*H*-pyrazolo[3,4-*d*]pyrimidine ring in **1a** occupied the ATP-binding pocket, and formed hydrogen bonds with the hinge region of BRAF^V600E^. The diaryl urea portion extended into the large hydrophobic back pocket created by the rearrangement of the DFG motif, and the urea CO and NH moiety formed a hydrogen bond with the backbone-NH of Asp 594 and side-chain carboxylate of Glu 501 in the DFG motif, respectively. These suggested that the diaryl urea contributes significantly to the inhibitor´s affinity. Considering the importance of the diaryl urea for the potency of kinase, we decided to maintain the diaryl urea portion in our inhibitors and tried to focus on the replacement of the terminal phenyl to investigate their effects on kinase inhibitory activities in this paper. In order to illustrate the effect of the N1 hydrogen on the inhibitory activity, N1-methylated compounds were also designed. Herein, we would like to report the synthesis and biological evaluation of these series compounds as novel pan-BRAF inhibitors. The molecular modelling study was also discussed in this paper.

## 2. Results

### 2.1. Chemistry

**Scheme 1:**
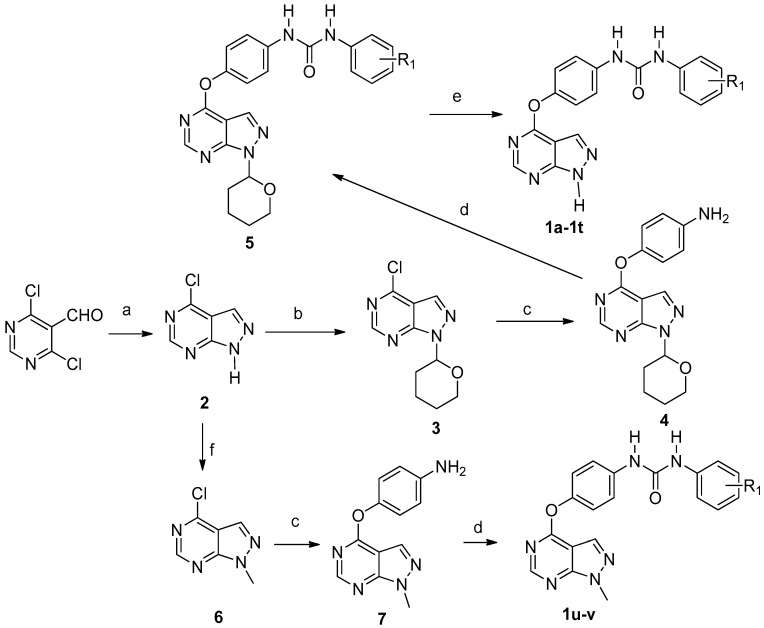
Synthetic route to compounds **1**. Reagents and conditions: (a) Hydrazine monohydrate, Et_3_N, MeOH, −65 °C, 2–3 h, 68.9%; (b) 3,4-Dihydro-2*H*-pyran, PPTs (pyridinium 4-toluenesulfonate), EtOAc, 50 °C, overnight, 76.5%; (c) 4-Aminophenol, Cs_2_CO_3_, DMF (dimethylformamide), N_2_, room temperature (r.t.), 79.5%; (d) Phenyl isocyanate, CH_2_Cl_2_, ice bath, overnight, 60.2–80.0%; (e) 4 M HCl/1,4-dioxane, r.t., overnight, 54.2–92.6%; (f) Iodomethane, NaH, DMF, 0 °C, 30 min, 69.5–70.8%.

The target compounds were prepared according to the synthetic routes outlined in Scheme 1, which was distinct from that described previously [22]. All target compounds were prepared from the key intermediate 4-chloro-1*H*-pyrazolo[3,4-*d*]pyrimidine (**2**). The preparation of **2** from allopurinol by chlorination was evaluated and determined to be unsuitable for large scale preparation due to poor stability of the product with respect to polymerization. We therefore synthesized compound **2** by cycloaddition reaction of 4,6-dichloropyrimidine-5-carbaldehyde and hydrazine monohydrate [23]. In order to avoid the side reaction of 1-NH of compound **2**, we designed the pyran-protected compound **3**. Compound **3** was synthesized from compound **2** and then nucleophilic substitution with 4-aminophenol in the present of Cs_2_CO_3_ gave the phenoxylated derivatives **4**. Condensation of **4** with the corresponding phenyl isocyanate in CH_2_Cl_2_ provided the urea **5** in moderate yields. Finally, compound **5** was deprotected using 4 M HCl in dioxane to produce the target compounds **1a**–**1t** in high yields. N1-methylated compounds **1u** and **1v** were also prepared from intermediate **2**. Compound **2** was methylated using iodomethane in the presence of sodium hydride to obtain compound **6**, with subsequent nucleophilic substitution with 4-aminophenol in the presence of Cs_2_CO_3_ to give the phenoxylated derivatives **7** in good yield. Finally, condensation with the corresponding phenyl isocyanate provided the target compounds **1u** and **1v** in high yields.

### 2.2. Kinase Inhibitory Activity

The enzyme activities are summarized in Table 1. Based on the results of molecular docking, we focused on optimization of the terminal phenyl ring to improve the kinase inhibitory activities. The effects of *ortho*-, *meta*-, and *para*-substitution and disubstitution on the terminal phenyl were investigated. Several trends can be observed from Table 1. The results displayed that the 4-chloro-3-trifluomethylphenyl group is important for potency against BRAF^V600E^, which is consistent with the molecular modelling that the terminal phenyl group in **1a** projects into a large hydrophobic pocket created by the rearrangement of the DFG motif. As we expected, compound **1e**, bearing no substitution on the terminal phenyl, displayed no inhibitory activity even at 1000 nM, which clearly indicated the importance of the hydrophobic interaction between the inhibitor and the hydrophobic amino acid residue of the back pocket. A small group of *ortho*-substitution such as fluoro is tolerated, whereas large groups are not tolerated. For example, compounds **1f** and **1r,** which bear 2-Cl and 2-NO_2_, are less potent than the parent compound **1b**. A variety of substituents at the *meta*-position, including both electron-donating and electron-withdrawing groups are tolerated, which suggested that the electronic interaction of the substituent had little effect on the kinase inhibitory activities. For example, introduction of electron-donating groups methyl and ethyl in compounds **1b** and **1l** increase the activities against BRAF^V600E^. Substitution with electron-withdrawing groups such as Cl and Br on the phenyl ring also leads to enhancement of kinase inhibitory activities (**1g** and **1s**). Substitution at the *para*-position is also tolerated. For example, compound **1c** with 4-chloro substitution is more potent than compound **1g**. In addition, di-substitution with two electron-withdrawing groups such as F, Cl and CF_3_ on the phenyl ring leads to dramatic improvement of kinase inhibitory activities (**1a**, **1c**, **1i**, **1j** and **1n**).

The modelling suggested that **1a** formed hydrogen bonds with the hinge region of BRAF^V600E^ and elaboration at the interaction at NH position might not be tolerated. Surprisingly, the N-methylation compounds **1u** and **1v** exhibited higher inhibitory activities against BRAF^V600E^ than the unsubstituted counterparts **1g** and **1a**, implying that the methyl substitution at the N1 position of 1*H*-pyrazolo[3,4-*d*]pyrimidine was beneficial for the inhibitory activity of BRAF^V600E^. The favorable hydrophobic interaction imposed by the N1-methyl might be responsible for the improvement in potency.

Subsequently, we further evaluated the inhibitory activities of compounds **1l**, **1u** and **1v**, which exhibited excellent inhibitory activities at 1000 nM and 100 nM (Table 2). As shown in Table 2, **1l**, **1u** and **1v** exhibited stronger inhibitory activities against BRAF^V600E^ than the positive control Sorafenib.

### 2.3. In Vitro Anti-Proliferative Activity

The cell growth inhibitory activities of newly synthesized compounds were evaluated against four human cancer cell lines, including wild-type BRAF cell lines (PC-3 and A549), and BRAF^V600E^ cell lines (A375, HT-29) using standard MTT (3-(4,5-dimethyl-2-thiazolyl)-2,5-diphenyl-2*H*-tetrazolium bromide) assay in vitro, with Sorafenib as the positive control (Table 3). Additionally, cytotoxic assays were conducted using normal cell line MDCK (Madin-Darby canine kidney). As depicted in Table 3, most compounds showed moderate to good anti-proliferative activities, while compounds **1e** and **1r** proved to be ineffective against all four cell lines (half maximal inhibitory concentration, IC_50_ > 80 μM). Some of the synthesized compounds exhibited similar anti-proliferative activities compared with Sorafenib against two or more cell lines. Remarkably, compounds **1c**, **1f**, **1h**, **1i**, **1o** and **1s** demonstrated much higher anti-proliferative activities than the positive control Sorafenib against the PC-3 cell line. In particular, compounds **1f** and **1i** represented a 12-fold improvement in activity compared to Sorafenib with the IC_50_ value of 1.12 μM. Meanwhile, compound **1f** exhibited a 2-fold improvement compared to Sorafenib in inhibiting A549 cell line proliferation. Compound **1n** displayed stronger anti-proliferative activities than Sorafenib against the A375 cell line. Unfortunately, the results indicated that most of the compounds were ineffective (IC_50_ > 80 μM) on the HT-29 cell line. In addition, the results demonstrated that compounds with two electron-withdrawing substituents on the terminal phenyl ring (**1a**, **1c**, **1i**, and **1n**) also presented promising anti-proliferative activities. It is worth noting that most compounds indicated no inhibitory activity against MDCK cell lines (IC_50_ > 80 μM), which suggested that the compounds exhibit good selectivity towards cancer cells rather normal cells.

### 2.4. Kinase Inhibitory Profiles of Compound ***1v***

Compound **1v** exhibited higher inhibitory activity against BRAF^V600E^ than positive control Sorafenib with IC_50_ values of 23.6 nM, effective cellular anti-proliferative potencies against four tumor cell lines (A375, HT-29, PC-3, A549) which were comparable to the efficacy of Sorafenib, as well as better selection to normal cells compared with Sorafenib. Therefore, we selected compound **1v** for further study. The kinase selectivity profile of compound **1v** was assessed over 19 different protein kinases (Table 4). The results exhibited that **1v** had potent inhibitory activity against not only BRAF^V600E^ (IC_50_ = 23.6 nM) but also wild-type BRAF (IC_50_ = 51.5 nM) and C-RAF (IC_50_ = 8.5 nM), and almost no significant inhibitory activity against 13 other tested protein kinases even at concentration of 1000 nM. The results revealed that compound **1v** had a very good selectivity profile and was a pan-RAF kinases inhibitor.

### 2.5. Flow Cytometric Analysis of Cell-Cycle Arrest

**Figure 4:**
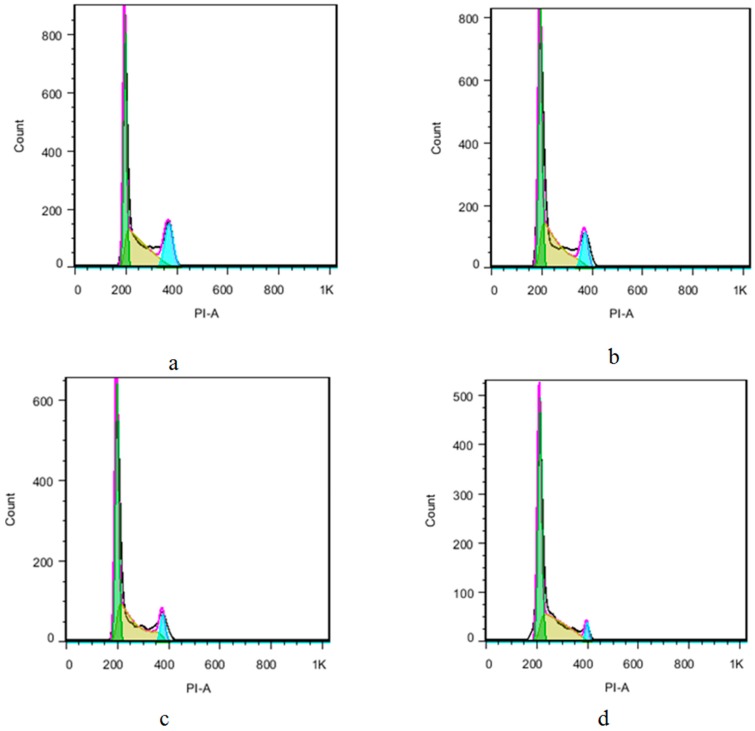
Flow cytometry analysis of cell cycle distribution in an A375 cell line treated with **1v** for 48 h. (**a**) Control; (**b**) 6.11 μmol/L (1/2 IC_50_); (**c**) 12.21 μmol/L (IC_50_); (**d**) 24.42 μmol/L (2 × IC_50_). PI-A: Propidium iodide-Annexin V.

To study the effect of the target compounds on cell cycle progression, a flow-activated cell sorting analysis was performed to examine the cell cycle progression upon treatment with the representative compound **1v** which exhibited most promising kinase inhibitory and anti-proliferative activities. As shown in Figure 4 and Table 5, cell cycle assay of the A375 cell line following treatment with **1v** showed a dose-dependent cell cycle arrest in the ‘G0/G1’ phase and a decrease in ‘G2/M’ phase of cell cycle in the treated group compared to the control.

### 2.6. Signaling Inhibition in A375 and HT-29 Cell Lines

**Figure 5:**
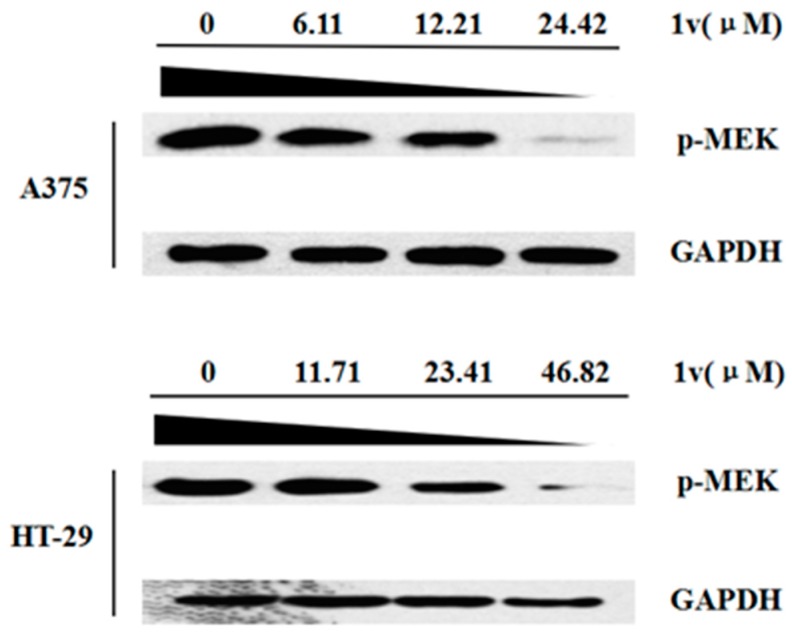
Mitogen-activated protein kinase (MAPK) signal suppression effect by 48 h treatment of **1v** (western blotting) in two cancer cell lines. GAPDH (glyceraldehyde-3-phosphate dehydrogenase) was used as a loading control. p-MEK: phosphorylated MAP kinase kinase.

To determine the potency of **1v** as a RAF inhibitor, we preliminary investigated the in vitro MAPK signal suppression effect of **1v** in A375 and HT-29 cell lines. The effect of compound **1v** on the expression of phosphor-MEK protein (RAF’s downstream target protein) in A375 and HT-29 cell lines was assessed by western blot analysis. As shown in Figure 5, **1v** suppressed phosphor-MEK levels in a concentration-dependent manner in two cancer cell lines, reflecting the inhibition of the downstream molecules in MAPK signal transduction. Notably, the inhibitory potency of compound **1v** on MEK phosphorylation was not so conspicuous at IC_50_ concentration, which may be related to the complicated mechanism of tumor cell growth and MEK phosphorylation. On one hand, the anti-proliferative IC_50_ values were determined by cell survival or proliferation, which is regulated by several different factors [24]. On the other hand, MEK phosphorylation is regulated by not only by RAF but also other kinases such as Mos (an upstream activator of mitogen-activated protein kinase) and MEKK-1 (mitogen activated protein kinase kinase kinase 1) [25,26]. When compound **1v** was applied at its anti-proliferative IC_50_ concentration, the expression of MEK phosphorylation via RAF regulation might have been just partly inhibited, and other kinases could still activate MEK phosphorylation, which resulted in the unobvious inhibitory effects of MEK phosphorylation. However, when the concentration reached twice the IC_50_, MEK phosphorylation was obviously inhibited, implying that the compound **1v** can effectively inhibit the Ras/RAF/MEK/ERK pathway at higher concentrations.

### 2.7. Molecular Modelling

**Figure 6:**
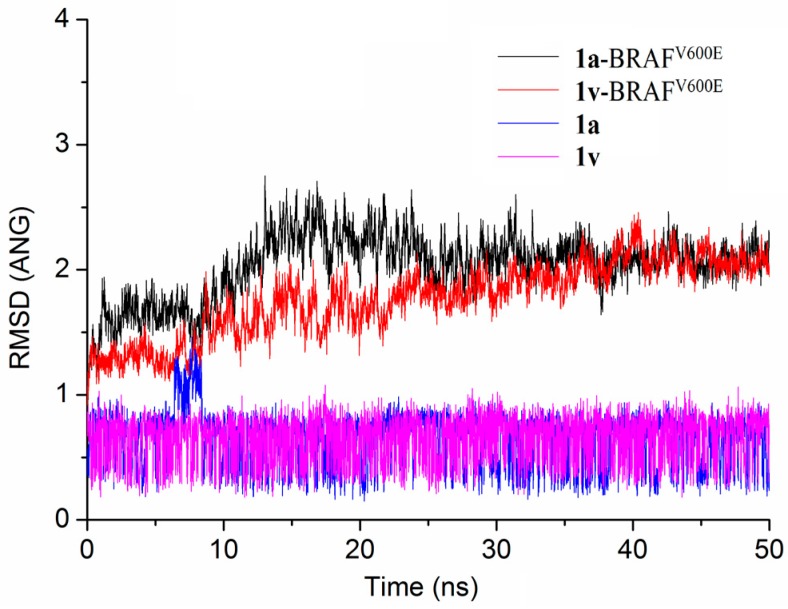
Root-mean-square deviations (RMSDs) of backbone atoms (C, Cα, and N) of protein, and the heavy atoms of the ligand for the simulated systems.

**Figure 7:**
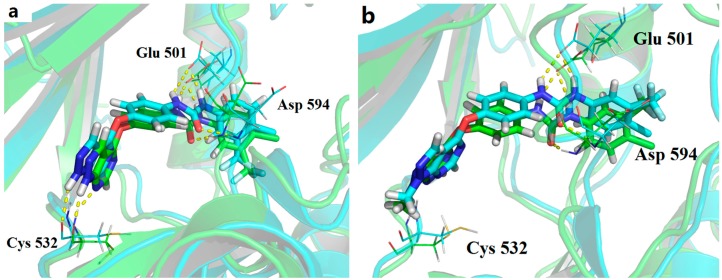
Binding mode of inhibitors within the active site of BRAF^V600E^. Structural comparison between docked (cyan) and molecular dynamics (MD)-simulated representative snapshots (green) of **1a** in the active conformation (**a**); **1v** in the active conformation (**b**). Yellow dots represent hydrogen bonds. (BRAF^V600E^ in colored ribbon, ligands in stick model, the key residues in line model).

In order to analyze in depth the interaction of synthesized compounds with BRAF, we performed a molecular dynamics (MD) study on **1a** and N1-methylated compound **1v** in complex with BRAF^V600E^. Firstly, compound **1v** was also successfully docked into the active site of BRAF^V600E^ (PDB code: 1UWJ) using Surflex–Dock. MD simulations were then carried out on inhibitors **1a** and **1v** in complex with BRAF^V600E^ in explicit aqueous solution for 50 ns. The stability of the system under simulation was evaluated by the root-mean-square deviation (RMSD) of the backbone atoms relating to the starting structures (Figure 6). As can be seen in the plot, all systems were stable during the 50 ns MD simulation. We compared the representative snapshots of each complex taken from the last 10 ns MD trajectories with the initial structure (Figure 7). As illustrated in Figure 7, the binding modes of **1a** and **1v** from MD simulated results were nearly the same as the docked structures. The 1*H*-pyrazolo[3,4-*d*]pyrimidine moiety inserted into the deep cleft of ATP-binding site, and the diaryl urea portion extended into the large hydrophobic back pocket created by the rearrangement of the DFG motif.

The hydrogen bond plays an important role in inhibitor binding to kinase. The major interactions were the important H-bonding interactions between inhibitors **1a** and **1v** and with Glu 501, Asp594 and Cys532 (Table 6). The CO and NH motifs of the urea group of compounds **1a** and **1v** formed hydrogen bond interactions with the backbone-NH of Asp594 and side-chain carboxylate of Glu 501 in the DFG motif with very high hydrogen-bond occupancies, respectively. The N7 of 1*H*-pyrazolo[3,4-*d*]pyrimidine of compounds **1a** and **1v** formed moderate hydrogen bond interactions with Cys532 in the hinge region of BRAF^V600E^. The 1*H*-pyrazolo[3,4-*d*]pyrimidine N1H of compound **1a** established a weak hydrogen bond interaction with backbone of Cys 532.

The binding free energies were calculated by using MM-PBSA (molecular mechanics Poisson–Boltzmann surface area) and MM-GBSA (molecular mechanics Generalized-Born surface area) programs in AMBER (Table 7). As can been seen, MM-PBSA and MM-GBSA calculations verified that ligand **1v** showed improved potency compared to that of **1a**, which was in good agreement with the experimental data. According to the energy individual components of the binding free energies, the favorable contributors to ligand binding were van der Waals (vdW) terms, electrostatic and nonpolar salvation energies, whereas polar solvation and entropy terms oppose binding. The favorable electrostatic interactions were counteracted by the unfavorable electrostatics of desolvation upon binding. Consequently, the total electrostatic interaction contributions were unfavorable to binding in all systems.

## 3. Materials and Methods

### 3.1. Chemistry

Melting points were determined using an X-4 Melting-point Apparatus with Microscope (Yuhua Instrument Co., Ltd., Henan, China) and were uncorrected. ^1^H- and ^13^C-NMR spectra were recorded on a Bruker AVIII 400 MHz or Bruker AVIII 600 MHz and 100 MHz spectrometer (Bruker Co., Fällanden, Switzerland) with tetramethylsilane (TMS) as the internal standard, the values of the chemical shifts (δ) are given in ppm, and coupling constants (*J*) are given in Hz. Mass spectra (ESI-MS) were performed on Waters ZQ4000 (Waters Co., Milford, MA, USA). All reactions were monitored using thin-layer chromatography (TLC) on silica gel plates at 254 nm under an ultraviolet (UV) light. Flash column chromatography separations were performed on normal phase silica gel (200–300 mesh, Merck Co., Elkton, VA, USA) or reverse phase silica gel by using Yamazen AI-580 flash chromatography (Yamazen Co., Osaka, Japan) with UV detection at 254 nm. All reagents were purchased from commercial vendors and were used without further purification. All oxygen-sensitive or moisture-sensitive reactions were run under nitrogen atmosphere. Yields were not optimized.

*4-Chloro-1H-pyrazolo[3,4-d]pyrimidine* (**2**). To the solution of 4,6-dichloropyrimidine-5-carbaldehyde (1.0 g, 0.006 mol) in methanol (20 mL) at −65 °C, triethylamine (0.97 mL,1.2 equivalents (eq.)) was added. A solution of hydrazine monohydrate (0.274 mL 1.0 eq.) in methanol (10 mL) was slowly dripped into above stirred solution by using a constant-pressure dropping funnel. The mixture was allowed to warm to room temperature and stirred for 2–3 h. The reaction mixture was concentrated in vacuo and crude product was diluted with water (20 mL), and extracted with EtOAc (60 mL × 3). The combined organic layer was washed with saturated solution of NaCl (60 mL × 3), dried over MgSO_4_ and concentrated to give compound **2**. Yield: 68.9%. ^1^H-NMR (400 MHz, deuteriated dimethyl sulfoxide (DMSO-*d*_6_)) δ 14.51 (s, 1H), 8.84 (s, 1H), 8.45 (s, 1H). ESI-MS *m*/*z*: 153.00 [M − H]^−^.

*4-Chloro-1-(tetrahydro-2H-pyran-2-yl)-1H-pyrazolo[3,4-d]pyrimidine* (**3**). Compound **2** (1.88 g, 0.012 mol) was dissolved in EtOAc (50 mL) and heated to 50 °C. After 10 min pyridinium 4-toluenesulfonate (PPTs) (50 mg) were added, followed by the addition of 3,4-dihydro-2*H*-pyran. The resulting reaction mixture was at stirred 50 °C. After the reaction was complete according to the TLC detection, the mixture was cooled to room temperature, washed with water (60 mL × 1), and a saturated solution of NaCl (50 mL × 2), and dried over MgSO_4_. The ethyl acetate was removed and petroleum ether (60 mL × 2) was added. The mixture was heated and filtered through cotton. Removal of petroleum ether in vacuo gives compound **3** as light yellow colored solid. Yield: 76.5%. ^1^H-NMR (400 MHz, DMSO-*d*_6_) δ 8.92 (s, 1H), 8.55 (s, 1H), 6.02 (dd, *J =* 10.4, 2.4 Hz, 1H), 3.97 (d, *J* = 12.0 Hz, 1H), 3.76–3.70 (m, 1H), 2.49–2.42 (m, 1H), 2.07–2.08 (m, 1H), 1.98–1.94 (m, 1H), 1.85–1.73 (m, 1H), 1.64–1.58 (m, 2H). ESI-MS *m*/*z*: 239.06 [M + H]^+^.

*4-((1-(Tetrahydro-2H-pyran-2-yl)-1H-pyrazolo[3,4-d]pyrimidin-4-yl)oxy)aniline* (**4**). To the mixture of *para*-aminophenol (0.28 g) in dimethylformamide (DMF, 2 mL), Cs_2_CO_3_ (1.2 eq.) was added under the protection of nitrogen. The reaction mixture was stirred at room temperature for 1.5–2 h, and then compound **3** was added slowly. After stirring overnight, the reaction mixture was diluted with EtOAc (50 mL) and washed with 1 M NaOH (60 mL × 1), water (80 mL × 1), 5% LiCl (50 mL × 2) and brine. The organic layer was dried over MgSO_4_ and concentrated to give compound **4** as a brown oily solid. Yield: 79.5%. ^1^H-NMR (400 MHz, DMSO-*d*_6_) δ 8.57 (s, 1H), 7.75 (s, 1H), 6.99–6.95 (m, 2H), 6.67–6.31 (m, 2H), 5.96 (dd, *J* = 10.0, 2.4 Hz, 1H), 5.19 (s, 2H), 3.96 (d, *J* = 12.4 Hz, 1H), 3.73–3.67 (m, 1H), 2.48–2.40 (m, 1H), 2.06–2.00 (m, 1H), 1.92–1.88 (m, 1H), 1.79–1.71 (m, 1H), 1.61–1.56 (m, 2H). ESI-MS *m*/*z*: 310.14 [M − H]^−^.

*1-(4-Chloro-3-(trifluoromethyl)phenyl)-3-(4-((1-(tetrahydro-2H-pyran-2-yl)-1H-pyrazolo[3,4-d]pyrimidin-4-yl)oxy)phenyl)urea* (**5a**). To the solution of compound **4** in CH_2_Cl_2_ at 0 °C 4-chloro-3-(trifluoromethyl)phenyl isocyanate (1.0 eq.) was added. The mixture was stirred overnight at room temperature. To the resulting suspension, petroleum ether (60 mL) was added. The solid material was collected by filtration to provide the title compound as a white solid. Yield: 66.6%. ^1^H-NMR (400 MHz, DMSO-*d*_6_) δ 9.22 (s, 1H), 8.99 (s, 1H), 8.58 (s, 1H), 8.14 (s, 2H), 7.69–7.57 (m, 4H), 7.27 (d, *J* = 8.8 Hz, 2H), 5.98 (d, *J* = 10.0 Hz, 1H), 3.97 (d, *J* = 11.6 Hz, 1H), 3.74–3.68 (m, 1H), 2.05 (d, *J* = 12.4 Hz, 1H), 1.93 (d, *J* = 12.4 Hz, 1H), 1.77 (d, *J =* 8.0 Hz, 1H), 1.59 (s, 3H). ^13^C-NMR (100 MHz, DMSO-*d*_6_) δ 163.7, 155.7, 152.9, 147.1, 139.8, 137.6, 132.4, 132.3, 127.3, 123.5, 122.7, 121.9, 120.3, 117.3, 102.7, 82.7, 67.5, 29.1, 25.0, 22.6. ESI-MS *m*/*z*: 533.12 [M + H]^+^.

*1-(4-((1-(Tetrahydro-2H-pyran-2-yl)-1H-pyrazolo[3,4-d]pyrimidin-4-yl)oxy)phenyl)-3-(m-tolyl)urea* (**5b**). Compound **5b** was prepared using the same procedure as described for the synthesis of **5a** by replacing 4-chloro-3-(trifluoromethyl)phenyl isocyanate with 3-methyl phenyl isocyanate. Yield: 80.0%. ^1^H-NMR (400 MHz, DMSO-*d*_6_) δ 14.13 (s, 1H), 8.82 (s, 1H), 8.66 (s, 1H), 8.51 (s, 1H), 8.01 (s, 1H), 7.56 (d, *J* = 8.9 Hz, 2H), 7.32 (s, 1H), 7.25 (d, *J* = 8.9 Hz, 3H), 7.17 (t, *J* = 7.7 Hz, 1H), 6.80 (d, *J* = 7.7 Hz, 1H), 2.29 (s, 3H). ^13^C-NMR (100 MHz, DMSO-*d*_6_) δ 163.68, 157.13, 155.39, 153.00, 146.77, 139.99, 138.36, 138.11, 132.26, 129.04, 123.02, 122.66, 120.47, 119.63, 117.67, 117.24, 102.01. ESI-MS *m*/*z*: 445.19 [M − H]^−^.

*1-(3,4-Dichlorophenyl)-3-(4-((1-(tetrahydro-2H-pyran-2-yl)-1H-pyrazolo[3,4-d]pyrimidin-4-yl)oxy)phenyl) urea* (**5c**). Compound **5c** was prepared using the same procedure as described for the synthesis of **5a** by replacing 4-chloro-3-(trifluoromethyl)phenyl isocyanate with 3,4-dichlorophenyl isocyanate. Yield: 67.0%. ^1^H-NMR (400 MHz, DMSO-*d*_6_) δ 9.07 (s, 1H), 8.97 (s, 1H), 8.58 (s, 1H), 8.14 (s, 1H), 7.90 (s, 1H), 7.58–7.52 (m, 3H), 7.37 (d, *J* = 8.4 Hz, 1H), 7.27 (d, *J* = 8.8 Hz, 2H), 5.99 (d, *J* = 9.6 Hz, 1H), 3.97 (d, *J* = 10.8 Hz, 1H), 3.71 (s, 1H), 2.05 (d, *J* = 12.8 Hz, 1H), 1.93 (d, *J* = 12.4 Hz, 1H), 1.79 (s, 1H), 1.59 (s, 2H), 1.24 (s, 1H). ESI-MS *m*/*z*: 499.10 [M + H]^+^.

*1-(4-Chlorophenyl)-3-(4-((1-(tetrahydro-2H-pyran-2-yl)-1H-pyrazolo[3,4-d]pyrimidin-4-yl)oxy)phenyl)urea* (**5d**). Compound **5d** was prepared using the same procedure as described for the synthesis of **5a** by replacing 4-chloro-3-(trifluoromethyl)phenyl isocyanate with 4-chlorophenyl isocyanate. Yield: 68.3%. ^1^H-NMR (400 MHz, DMSO-*d*_6_) δ 8.87 (s, 1H), 8.85 (s, 1H), 8.58 (s, 1H), 8.13 (s, 1H), 7.56 (d, *J* = 8.9 Hz, 2H), 7.51 (d, *J* = 8.8 Hz, 2H), 7.26 (d, *J* = 8.8 Hz, 2H), 7.26 (d, *J* = 8.9 Hz, 2H), 5.98 (dd, *J* = 10.1, 1.9 Hz, 1H), 3.97 (d, *J* = 11.2 Hz, 1H), 3.77–3.65 (m, 1H), 2.49–2.41 (m, 1H), 2.05 (d, *J* = 12.5 Hz, 1H), 1.93 (dd, *J* = 12.9, 2.3 Hz, 1H), 1.83–1.68 (m, 1H), 1.67–1.53 (m, 2H). ESI-MS *m*/*z*: 465.14 [M + H]^+^.

*1-Phenyl-3-(4-((1-(tetrahydro-2H-pyran-2-yl)-1H-pyrazolo[3,4-d]pyrimidin-4-yl)oxy)phenyl)urea* (**5e**). Compound **5e** was prepared using the same procedure as described for the synthesis of **5a** by replacing 4-chloro-3-(trifluoromethyl)phenyl isocyanate with phenyl isocyanate. Yield: 60.2%. ^1^H-NMR (400 MHz, DMSO-*d*_6_) δ 8.81 (s, 1H), 8.71 (s, 1H), 8.58 (s, 1H), 8.12 (s, 1H), 7.57 (d, *J* = 7.7 Hz, 2H), 7.48 (d, *J* = 7.7 Hz, 2H), 7.33–7.28 (t, 2H), 7.26 (d, *J* = 8.9 Hz, 2H), 6.99 (t, *J* = 7.3 Hz, 1H), 5.99 (d, *J* = 12.5 Hz, 1H), 3.97 (d, *J* = 11.2 Hz, 1H), 3.76–3.66 (m, 1H), 2.45 (m, 1H), 2.03 (m, 1H), 1.93 (m, 1H), 1.86–1.69 (m, 1H), 1.66–1.53 (m, 2H). ESI-MS *m*/*z*: 431.18 [M + H]^+^.

*1-(2-Chloro-5-methylphenyl)-3-(4-((1-(tetrahydro-2H-pyran-2-yl)-1H-pyrazolo[3,4-d]pyrimidin-4-yl)oxy) phenyl)urea* (**5f**). Compound **5f** was prepared using the same procedure as described for the synthesis of **5a** by replacing 4-chloro-3-(trifluoromethyl)phenyl isocyanate with 2-chloro-5-methylphenyl isocyanate. Yield: 69.4%. ^1^H-NMR (400 MHz, DMSO-*d*_6_) δ 9.54 (s, 1H), 8.59 (s, 1H), 8.28 (s, 1H), 8.13 (s, 1H), 8.03 (s, 1H), 7.58 (d, *J* = 8.8 Hz, 2H), 7.34 (d, *J* = 8.0 Hz, 1H), 7.28 (d, *J =* 8.8 Hz, 2H), 6.87 (d, *J =* 8.0 Hz, 1H), 5.99 (d, *J =* 10.0 Hz, 1H), 3.97 (d, *J =* 11.2 Hz, 1H), 3.75–3.68 (m, 1H), 2.30 (s, 3H), 2.08–2.00 (m, 1H), 1.93 (d, *J =* 11.6 Hz, 1H), 1.77 (s, 1H), 1.60 (s, 2H), 1.24 (s, 1H). ESI-MS *m*/*z*: 477.15 [M − H]^−^.

*1-(3-Chlorophenyl)-3-(4-((1-(tetrahydro-2H-pyran-2-yl)-1H-pyrazolo[3,4-d]pyrimidin-4-yl)oxy)phenyl)urea* (**5g**). Compound **5g** was prepared using the same procedure as described for the synthesis of **5a** by replacing 4-chloro-3-(trifluoromethyl)phenyl isocyanate with 3-chlorophenyl isocyanate. Yield: 72.3%. ^1^H-NMR (400 MHz, DMSO-*d*_6_) δ 8.94 (s, 1H), 8.90 (s, 1H), 8.60 (s, 1H), 8.14 (s, 1H), 7.73 (s, 1H), 7.57 (d, *J =* 9.0 Hz, 2H), 7.30 (m, 5H), 7.03 (m, 1H), 5.99 (dd, *J =* 10.2, 2.3 Hz, 1H), 3.97 (d, *J =* 12.0 Hz, 1H), 3.76–3.66 (m, 1H), 2.45 (m, 1H), 2.05 (m, 1H), 1.93 (m, 1H), 1.83–1.72 (m, 1H), 1.61 (m, 2H). ESI-MS *m*/*z*: 463.14 [M − H]^−^.

*1-(2,3-Dimethylphenyl)-3-(4-((1-(tetrahydro-2H-pyran-2-yl)-1H-pyrazolo[3,4-d]pyrimidin-4-yl)oxy)phenyl) urea* (**5h**). Compound **5h** was prepared using the same procedure as described for the synthesis of **5a** by replacing 4-chloro-3-(trifluoromethyl)phenyl isocyanate with 2,3-dimethylphenyl isocyanate. Yield: 65.5%. ^1^H-NMR (400 MHz, DMSO-*d*_6_) δ 9.07 (s, 1H), 8.58 (s, 1H), 8.10 (s, 1H), 8.00 (s, 1H), 7.56 (t, *J =* 8.6 Hz, 3H), 7.25 (d, *J =* 8.4 Hz, 1H), 7.05 (t, *J =* 7.8 Hz, 1H), 6.92 (d, *J =* 7.2 Hz, 1H), 5.99 (d, *J =* 9.2 Hz, 1H), 3.97 (d, *J =* 11.2 Hz, 1H), 3.75–3.68 (m, 1H), 2.45 (d, *J =* 10.4 Hz, 1H), 2.27 (s, 3H), 2.16 (s, 3H), 2.05 (d, *J =* 10.8 Hz, 1H), 1.93 (d, *J =* 12.0 Hz, 1H), 1.79 (s, 1H), 1.60 (s, 2H), 1.46 (s, 1H). ESI-MS *m*/*z*: 481.21 [M + Na]^+^.

*1-(2-Chloro-5-(trifluoromethyl)phenyl)-3-(4-((1-(tetrahydro-2H-pyran-2-yl)-1H-pyrazolo[3,4-d]pyrimidin-4-yl)oxy)phenyl)urea* (**5i**). Compound **5i** was prepared using the same procedure as described for the synthesis of **5a** by replacing 4-chloro-3-(trifluoromethyl)phenyl isocyanate with 2-chloro-5-(trifluoromethyl)phenyl isocyanate. Yield: 74.5%. ^1^H-NMR (400 MHz, DMSO-*d*_6_) δ 9.22 (s, 1H), 8.99 (s, 1H), 8.58 (s, 1H), 8.14 (s, 2H), 7.69–7.57 (m, 4H), 7.27 (d, *J =* 8.8 Hz, 2H), 5.98 (d, *J =* 10.0 Hz, 1H), 3.97 (d, *J =* 11.6 Hz, 1H), 3.74–3.68 (m, 1H), 2.05 (d, *J =* 12.4 Hz, 1H), 1.93 (d, *J =* 12.4 Hz, 1H), 1.77 (d, *J =* 8.0 Hz, 1H), 1.59 (s, 3H). ESI-MS *m*/*z*: 533.12 [M + H]^+^.

*1-(3-Fluoro-5-(trifluoromethyl)phenyl)-3-(4-((1-(tetrahydro-2H-pyran-2-yl)-1H-pyrazolo[3,4-d]pyrimidin-4-yl)oxy)phenyl)urea* (**5j**). Compound **5j** was prepared using the same procedure as described for the synthesis of **5a** by replacing 4-chloro-3-(trifluoromethyl)phenyl isocyanate with 3-fluoro-5-(trifluoromethyl)phenyl isocyanate. Yield: 60.2%. ^1^H-NMR (400 MHz, DMSO-*d*_6_) δ 9.31 (s, 1H), 9.06 (s, 1H), 8.58 (s, 1H), 8.15 (s, 1H), 7.73 (s, 1H), 7.64 (d, *J =* 11.2 Hz, 1H), 7.58 (d, *J =* 8.8 Hz, 2H), 7.28 (d, *J =* 8.8 Hz, 2H), 7.24 (d, *J =* 8.5 Hz, 1H), 5.99 (dd, *J =* 10.1, 2.1 Hz, 1H), 3.97 (d, *J =* 11.6 Hz, 1H), 3.76–3.66 (m, 1H), 2.45 (dd, *J =* 12.8, 4.0 Hz, 1H), 2.05 (d, *J =* 12.5 Hz, 1H), 1.93 (dd, *J =* 13.0, 2.6 Hz, 1H), 1.84–1.70 (m, 1H), 1.61 (d, *J =* 10.0 Hz, 2H). ESI-MS *m*/*z*: 517.15 [M + H]^+^.

*1-(4-Fluoro-3-methylphenyl)-3-(4-((1-(tetrahydro-2H-pyran-2-yl)-1H-pyrazolo[3,4-d]pyrimidin-4-yl)oxy)phenyl)urea* (**5k**). Compound **5k** was prepared using the same procedure as described for the synthesis of **5a** by replacing 4-chloro-3-(trifluoromethyl)phenyl isocyanate with 4-fluoro-3-methylphenyl isocyanate. Yield: 79.4%. ^1^H-NMR (400 MHz, DMSO-*d*_6_) δ 8.83 (s, 1H), 8.69 (s, 1H), 8.58 (s, 1H), 8.11 (s, 1H), 7.56 (d, *J =* 8.8 Hz, 2H), 7.38 (dd, *J =* 6.8, 2.4 Hz,1H), 7.30–7.24 (m, 3H), 7.06 (t, *J =* 9.2 Hz, 1H), 5.98 (dd, *J =* 10.4, 2.4 Hz, 1H), 3.97 (d, *J =* 10.8 Hz, 1H), 3.74–3.66 (m, 1H), 2.22 (s, 3H), 2.05 (d, *J =* 11.6 Hz,1H), 1.95–1.90 (m, 1H), 1.80–1.76 (m, 1H), 1.59 (s, 2H), 1.24 (s, 1H). ESI-MS *m*/*z*: 485.15 [M + Na]^+^.

*1-(3-Ethylphenyl)-3-(4-((1-(tetrahydro-2H-pyran-2-yl)-1H-pyrazolo[3,4-d]pyrimidin-4-yl)oxy)phenyl)urea* (**5l**). Compound **5l** was prepared using the same procedure as described for the synthesis of **5a** by replacing 4-chloro-3-(trifluoromethyl)phenyl isocyanate with 3-ethylpheny isocyanate. Yield: 69.4%. ^1^H-NMR (400 MHz, DMSO-*d*_6_) δ 8.79 (s, 1H), 8.65 (s, 1H), 8.58 (s, 1H), 8.11 (s, 1H), 7.56 (d, *J =* 8.9 Hz, 2H), 7.37 (d, *J =* 9.2 Hz, 1H), 7.29 (s, 1H), 7.25 (d, *J =* 8.8 Hz, 2H), 7.06 (t, *J =* 9.2 Hz, 1H), 5.98 (dd, *J =* 9.9, 1.4 Hz, 1H), 3.97 (d, *J =* 11.4 Hz, 1H), 3.72 (m, 1H), 2.50–2.41 (m, 1H), 2.23 (s, 3H), 2.05 (m, 1H), 1.92 (m, 1H), 1.85–1.70 (m, 1H), 1.59 (m, 2H). ESI-MS *m*/*z*: 459.21 [M + H]^+^.

*1-(3-Nitrophenyl)-3-(4-((1-(tetrahydro-2H-pyran-2-yl)-1H-pyrazolo[3,4-d]pyrimidin-4-yl)oxy)phenyl)urea* (**5m**). Compound **5m** was s prepared using the same procedure as described for the synthesis of **5a** by replacing 4-chloro-3-(trifluoromethyl)phenyl isocyanate with 3-nitrophenyl isocyanate. Yield: 62.3%. ^1^H-NMR (400 MHz, DMSO-*d*_6_) δ 9.41 (s, 1H), 9.11 (s, 1H), 8.59 (s, 2H), 8.16 (s, 1H), 7.85 (s, 1H), 7.74 (s, 1H), 7.59 (s, 2H), 7.29–7.00 (m, 2H), 5.99 (d, *J =* 12.4 Hz, 1H), 5.31 (s, 1H), 3.96 (s, 2H), 3.72 (s, 2H), 2.02 (s, 2H), 1.77 (s, 1H), 1.59 (s, 1H). ^13^C-NMR (100 MHz, DMSO-*d*_6_) δ 167.36, 163.69, 155.70, 152.91, 148.58, 147.07, 141.45, 137.66, 132.23, 131.95, 130.46, 129.04, 124.77, 122.64, 120.27, 116.72, 112.61, 102.74, 82.72, 67.82, 28.77, 22.79. ESI-MS *m*/*z*: 474.16 [M − H]^−^.

*1-(2-Fluoro-5-(trifluoromethyl)phenyl)-3-(4-((1-(tetrahydro-2H-pyran-2-yl)-1H-pyrazolo[3,4-d]pyrimidin-4-yl)oxy)phenyl)urea* (**5n**). Compound **5n** was prepared using the same procedure as described for the synthesis of **5a** by replacing 4-chloro-3-(trifluoromethyl)phenyl isocyanate with 2-fluoro-5-(trifluoromethyl)phenyl isocyanate. Yield: 70.2%. ^1^H-NMR (400 MHz, DMSO-*d*_6_) δ 9.35 (s, 1H), 8.96 (s, 1H), 8.61 (d, *J =* 22.8 Hz, 2H), 8.16 (s, 1H), 7.55 (d, *J =* 26.4 Hz, 3H), 7.41 (s, 1H), 7.29 (d, *J =* 5.6 Hz, 2H), 5.98 (d, *J =* 8.8 Hz, 1H), 3.95 (s, 1H), 3.71 (s, 1H), 2.03 (s, 1H), 1.92 (d, *J =* 12.4 Hz, 2H), 1.78 (s, 1H), 1.59 (s, 2H). ESI-MS *m*/*z*: 517.15 [M + H]^+^.

*1-(3-(Methylthio)phenyl)-3-(4-((1-(tetrahydro-2H-pyran-2-yl)-1H-pyrazolo[3,4-d]pyrimidin-4-yl)oxy)phenyl)urea* (**5o**). Compound **5o** was prepared using the same procedure as described for the synthesis of **5a** by replacing 4-chloro-3-(trifluoromethyl)phenyl isocyanate with 3-(methylthio) phenyl isocyanate. Yield: 67.8%. ^1^H-NMR (400 MHz, DMSO-*d*_6_) δ 8.83 (s, 1H), 8.77 (s, 1H), 8.58 (s, 1H), 8.12 (s, 1H), 7.57 (d, *J =* 9.0 Hz, 2H), 7.49 (t, *J =* 1.7 Hz, 1H), 7.26 (d, *J =* 8.9 Hz, 2H), 7.22 (d, *J =* 7.8 Hz, 1H), 7.17 (d, *J =* 8.8 Hz, 1H), 6.88 (d, *J =* 7.8 Hz, 1H), 5.98 (dd, *J =* 10.2, 2.2 Hz, 2H), 2.51 (d, *J =* 1.8 Hz, 2H), 2.47 (s, 2H), 1.99 (dd, *J =* 42.9, 17.3 Hz, 2H), 1.59 (s, 3H). ESI-MS *m*/*z*: 477.16 [M + H]^+^.

*1-(3-Cyanophenyl)-3-(4-((1-(tetrahydro-2H-pyran-2-yl)-1H-pyrazolo[3,4-d]pyrimidin-4-yl)oxy)phenyl)urea* (**5p**). Compound **5p** was prepared using the same procedure as described for the synthesis of **5a** by replacing 4-chloro-3-(trifluoromethyl)phenyl isocyanate with 3-cyanophenyl isocyanate. Yield: 69.8%. ^1^H-NMR (400 MHz, DMSO-*d*_6_) δ 9.10 (s, 1H), 9.00 (s, 1H), 8.58 (s, 1H), 8.13 (s, 1H), 8.00 (t, *J =* 1.8 Hz, 1H), 7.71 (ddd, *J =* 3.2, 2.0, 1.2 Hz, 1H), 7.59 (d, *J =* 2.4 Hz, 1H), 7.57 (d, *J =* 2.0 Hz, 1H), 7.51 (t, *J =* 8.0 Hz, 1H), 7.43 (dt, *J =* 7.6, 1.2 Hz, 1H), 7.29–7.27 (m, 2H), 5.99 (dd, *J =* 10.0, 2.4 Hz, 1H), 3.18 (d, *J =* 5.2 Hz, 2H), 1.99 (s, 6H). ESI-MS *m*/*z*: 456.17 [M + H]^+^.

*Methyl 3-(3-(4-((1-(tetrahydro-2H-pyran-2-yl)-1H-pyrazolo[3,4-d]pyrimidin-4-yl)oxy)phenyl)ureido)benzoate* (**5q**). Compound **5q** was prepared using the same procedure as described for the synthesis of **5a** by replacing 4-chloro-3-(trifluoromethyl)phenyl isocyanate with methyl 3-isocyanatobenzoate. Yield: 74.5%. ^1^H-NMR (400 MHz, DMSO-*d*_6_) δ 9.03 (s, 1H), 8.89 (s, 1H), 8.58 (s, 1H), 8.23 (s, 1H), 8.13 (s, 1H), 7.65 (d, *J =* 7.9 Hz, 1H), 7.58 (d, *J =* 8.9 Hz, 3H), 7.44 (t, *J =* 7.9 Hz, 1H), 7.27 (d, *J =* 8.9 Hz, 2H), 5.98 (dd, *J =* 10.2, 2.1 Hz, 1H), 4.03–3.93 (m, 1H), 3.87 (s, 3H), 3.68–3.71 (m, 1H), 2.44–2.51 (m, 1H), 1.99–2.03 (m, 1H), 1.91–1.95 (m, 1H), 1.78 (m, 1H), 1.59 (m, 3H). ESI-MS *m*/*z*: 489.18 [M + H]^+^.

*1-(5-Methyl-2-nitrophenyl)-3-(4-((1-(tetrahydro-2H-pyran-2-yl)-1H-pyrazolo[3,4-d]pyrimidin-4-yl)oxy) phenyl)urea* (**5r**). Compound **5r** was prepared using the same procedure as described for the synthesis of **5a** by replacing 4-chloro-3-(trifluoromethyl)phenyl isocyanate with 5-methyl-2-nitrophenylisocyanate. Yield: 66.8%. ^1^H-NMR (400 MHz, DMSO-*d*_6_) δ 10.04 (s, 1H), 9.71 (s, 1H), 8.57 (s, 1H), 8.23 (s, 1H), 8.13 (s, 1H), 8.03 (d, *J =* 7.7 Hz, 1H), 7.61 (d, *J =* 6.9 Hz, 2H), 7.28 (d, *J =* 6.7 Hz, 2H), 7.02 (d, *J =* 7.3 Hz, 1H), 5.98 (d, *J =* 9.1 Hz, 1H), 3.96 (d, *J =* 10.6 Hz, 1H), 3.69 (d, *J =* 9.5 Hz, 1H), 2.39 (s, 3H), 1.98 (dd, *J =* 50.4, 13.4 Hz, 2H), 1.42 (d, *J =* 31.1 Hz, 2H), 0.84 (s, 2H). ESI-MS *m*/*z*: 490.18 [M + H]^+^.

*1-(3-Bromophenyl)-3-(4-((1-(tetrahydro-2H-pyran-2-yl)-1H-pyrazolo[3,4-d]pyrimidin-4-yl)oxy)phenyl)urea* (**5s**). Compound **5s** was prepared using the same procedure as described for the synthesis of **5a** by replacing 4-chloro-3-(trifluoromethyl)phenyl isocyanate with 3-bromophenylisocyanate. Yield: 79.0%. ^1^H-NMR (400 MHz, DMSO-*d*_6_) δ 8.92 (s, 1H), 8.89 (s, 1H), 8.58 (s, 1H), 8.13 (s, 1H), 7.87 (s, 1H), 7.57 (d, *J =* 8.8 Hz, 2H), 7.34 (d, *J =* 8.1 Hz, 1H), 7.28 (s, 1H), 7.24 (d, *J =* 8.7 Hz, 2H), 7.16 (d, *J =* 7.7 Hz, 1H), 5.98 (d, *J =* 8.8 Hz, 1H), 3.97 (d, *J =* 11.2 Hz, 1H), 3.71 (t, *J =* 12.6 Hz, 1H), 1.99 (dd, *J =* 47.7, 13.1 Hz, 2H), 1.82–1.71 (m, 1H), 1.59 (d, *J =* 3.0 Hz, 2H), 1.45–1.35 (m, 1H). ESI-MS *m*/*z*: 509.09 [M + H]^+^.

*1-(3,4-Dimethylphenyl)-3-(4-((1-(tetrahydro-2H-pyran-2-yl)-1H-pyrazolo[3,4-d]pyri-midin-4-yl)oxy) phenyl)urea* (**5t**). Compound **5t** was prepared using the same procedure as described for the synthesis of **5a** by replacing 4-chloro-3-(trifluoromethyl)phenyl isocyanate with 3,4-dimethylphenylisocyanate. Yield: 61.8%. ^1^H-NMR (400 MHz, DMSO-*d*_6_) δ 8.74 (s, 1H), 8.58 (s, 1H), 8.52 (s, 1H), 8.10 (s, 1H), 7.55 (d, *J =* 8.8 Hz, 2H), 7.25 (s, 2H), 7.23 (s, 1H), 7.19 (d, *J =* 8.1 Hz, 1H), 7.04 (d, *J =* 8.2 Hz, 1H), 5.98 (d, *J =* 9.7 Hz, 1H), 3.97 (dd, *J =* 10.9, 1.0 Hz, 1H), 3.75–3.66 (m, 1H), 2.44 (dd, *J =* 12.8, 3.9 Hz, 1H), 2.20 (s, 3H), 2.16 (s, 3H), 2.08–2.01 (m, 1H), 1.92 (d, *J =* 14.7 Hz, 1H), 1.83–1.73 (m, 1H), 1.59 (t, *J =* 8.3 Hz, 2H). ESI-MS *m*/*z*: 459.21 [M + H]^+^.

*1-(4-((1H-Pyrazolo[3,4-d]pyrimidin-4-yl)oxy)phenyl)-3-(4-chloro-3-(trifluoromet-hyl)phenyl)urea* (**1a**). A mixture of **1a** (0.1 mmol) in 4 M HCl/1,4-dioxane (5 mL) was stirred at room temperature for 30–70 min. To the resulting suspension, ether (60 mL) was added. The solid material was filtered, washed twice with ether and further purified by flash column chromatography to provide the title compound. Yield: 70.2%; m.p. 222.6–225.8 °C; ^1^H-NMR (400 MHz, DMSO-*d*_6_) δ 14.13 (s, 1H), 9.25 (s, 1H), 9.01 (s, 1H), 8.51 (s, 1H), 8.13 (s, 1H), 8.04 (s, 1H), 7.71–7.60 (m, 2H), 7.58 (d, *J =* 8.8 Hz, 2H), 7.27 (d, *J =* 8.8 Hz, 2H). ^13^C-NMR (100 MHz, DMSO-*d*_6_) δ 163.61, 157.13, 155.36, 152.91 147.18, 139.77, 137.54, 132.41, 132.25, 127.28, 126.98, 124.76, 123.48, 122.71, 120.31, 117.23, 101.76. ESI-MS *m*/*z*: 449.40 [M + H]^+^.

*1-(4-((1H-Pyrazolo[3,4-d]pyrimidin-4-yl)oxy)phenyl)-3-(m-tolyl)urea* (**1b**). Compound **1b** was prepared using the same procedure as described for the synthesis of **1a**. Yield: 71.8%; m.p. 227.4–230.0 °C; ^1^H-NMR (400 MHz, DMSO-*d*_6_) δ 14.13 (s, 1H), 8.82 (s, 1H), 8.66 (s, 1H), 8.51 (s, 1H), 8.01 (s, 1H), 7.56 (d, *J =* 8.9 Hz, 2H), 7.32 (s, 1H), 7.25 (d, *J =* 8.9 Hz, 3H), 7.17 (t, *J =* 7.7 Hz, 1H), 6.80 (d, *J =* 7.7 Hz, 1H), 2.29 (s, 3H). ^13^C-NMR (100 MHz, DMSO-*d*_6_) δ 163.76, 157.15, 155.33, 153.15, 148.13, 146.61, 140.15, 138.32, 138.29, 132.05, 128.99, 122.83, 122.62, 119.42, 118.90, 115.59, 101.75, 66.74, 21.63. ESI-MS *m*/*z*: 361.60 [M + H]^+^.

*1-(4-((1H-Pyrazolo[3,4-d]pyrimidin-4-yl)oxy)phenyl)-3-(3,4-dichlorophenyl)urea* (**1c**). Compound **1c** was prepared using the same procedure as described for the synthesis of **1a**. Yield: 92.6%; m.p. 245.0–247.2 °C; ^1^H-NMR (400 MHz, DMSO-*d*_6_) δ 14.12 (s, 1H), 9.08 (s, 1H), 8.97 (s, 1H), 8.51 (s, 1H), 8.03 (s, 1H), 7.90 (d, *J* = 2.4 Hz, 1H), 7.54 (dd, *J* = 27.2, 8.8 Hz, 3H), 7.36 (dd, *J* = 8.8, 2.4 Hz, 1H), 7.26 (d, *J* = 8.8 Hz, 2H). ^13^C-NMR (100 MHz, DMSO-*d*_6_) δ 163.59, 157.10, 155.34, 152.79, 147.08, 140.35, 137.60, 132.23, 131.43, 130.95, 123.53, 122.69, 120.14, 119.71, 118.76, 101.75. ESI-MS *m*/*z*: 415.04 [M + H]^+^.

*1-(4-((1H-Pyrazolo[3,4-d]pyrimidin-4-yl)oxy)phenyl)-3-(4-chlorophenyl)urea* (**1d**). Compound **1d** was prepared using the same procedure as described for the synthesis of **1a**. Yield: 80.5%; m.p. 260.8–262.0 °C; ^1^H-NMR (400 MHz, DMSO-*d*_6_) δ 14.13 (s, 1H), 8.86 (d, *J* = 10.2 Hz, 2H), 8.51 (s, 1H), 8.03 (s, 1H), 7.56 (d, *J* = 8.4 Hz, 2H), 7.51 (d, *J* = 8.8 Hz, 2H), 7.34 (d, *J* = 8.8 Hz, 2H), 7.26 (d, *J* = 8.4 Hz, 2H). ^13^C-NMR (100 MHz, DMSO-*d*_6_) δ 157.13, 155.38, 152.92, 146.92, 139.10, 137.88, 132.26, 129.04, 125.79, 122.69, 120.18, 119.94, 101.76. ESI-MS *m*/*z*: 391.50 [M + H]^+^.

*1-(4-((1H-Pyrazolo[3,4-d]pyrimidin-4-yl)oxy)phenyl)-3-phenylurea* (**1e**). Compound **1e** was prepared using the same procedure as described for the synthesis of **1a**. Yield: 81.2%; m.p. 247.3–249.2 °C; ^1^H-NMR (400 MHz, DMSO-*d*_6_) δ 14.13 (s, 1H), 8.80 (s, 1H), 8.71 (s, 1H), 8.52 (s, 1H), 8.02 (s, 1H), 7.56 (d, *J =* 8.9 Hz, 2H), 7.48 (d, *J =* 7.7 Hz, 2H), 7.30 (t, *J =* 7.7 Hz, 2H), 7.25 (d, *J =* 8.9 Hz, 2H), 6.98 (t, *J =* 7.3 Hz, 1H). ^13^C-NMR (100 MHz, DMSO-*d*_6_) δ 163.68, 157.13, 155.39, 153.01, 146.80, 140.07, 138.07, 132.27, 129.20, 122.68, 122.28, 119.79, 118.66, 101.76, 0.51. ESI-MS *m*/*z*: 347.40 [M + H]^+^.

*1-(4-((1H-Pyrazolo[3,4-d]pyrimidin-4-yl)oxy)phenyl)-3-(2-chloro-5-methylphenyl)urea* (**1f**). Compound **1f** was prepared using the same procedure as described for the synthesis of **1a**. Yield: 79.5%; m.p. 230.2–232.4 °C; ^1^H-NMR (400 MHz, DMSO-*d*_6_) δ 14.13 (s, 1H), 9.53 (s, 1H), 8.52 (s, 1H), 8.28 (s, 1H), 8.03 (s, 1H), 7.58 (d, *J* = 9.2 Hz, 1H), 7.34 (d, *J* = 8.0 Hz, 1H), 7.27 (d, *J* = 8.8 Hz, 2H), 6.87 (d, *J* = 9.2 Hz, 1H), 2.90 (s, 1H), 2.74 (s, 1H), 2.30 (s, 3H). ^13^C-NMR (100 MHz, DMSO-*d*_6_) δ 163.64, 157.14, 155.39, 152.60, 147.00, 137.81, 137.48, 135.95, 132.27, 129.22, 124.46, 122.79, 122.20, 119.75, 119.44, 101.76, 21.33, 0.51. ESI-MS *m*/*z*: 395.30 [M + H]^+^.

*1-(4-((1H-Pyrazolo[3,4-d]pyrimidin-4-yl)oxy)phenyl)-3-(3-chlorophenyl)urea* (**1g**). Compound **1g** was prepared using the same procedure as described for the synthesis of **1a**. Yield: 75.4%; m.p. 234.8–236.0 °C; ^1^H-NMR (400 MHz, DMSO-*d*_6_) δ 14.13 (s, 1H), 8.94 (s, 1H), 8.89 (s, 1H), 8.51 (s, 1H), 8.03 (s, 1H), 7.73 (s, 1H), 7.57 (d, *J =* 8.8 Hz, 2H), 7.34–7.26 (m, 4H), 7.06–7.00 (m, 1H). ^13^C-NMR (100 MHz, DMSO-*d*_6_) δ 163.64, 157.13, 155.37, 152.87, 147.01, 141.66, 137.75, 133.61, 132.26, 130.80, 122.70, 121.89, 120.04, 118.02, 117.10, 101.76, 99.93. ESI-MS *m*/*z*: 381.08 [M + H]^+^.

*1-(4-((1H-Pyrazolo[3,4-d]pyrimidin-4-yl)oxy)phenyl)-3-(2,3-dimethylphenyl)urea* (**1h**). Compound **1h** was prepared using the same procedure as described for the synthesis of **1a**. Yield: 81.4%; m.p. 250.4–252.3 °C; ^1^H-NMR (400 MHz, DMSO-*d*_6_) δ 14.13 (s, 1H), 9.06 (s, 1H), 8.51 (s, 1H), 8.00 (s, 2H), 7.57 (d, *J =* 6.8 Hz, 3H), 7.25 (d, *J =* 7.4 Hz, 2H), 7.06–7.02 (m, 1H), 6.91 (d, *J =* 6.6 Hz, 1H), 2.27 (s, 3H), 2.16 (s, 3H). ^13^C-NMR (100 MHz, DMSO-*d*_6_) δ 163.82, 157.13, 155.39, 153.40, 146.63, 138.40, 137.34, 137.00, 132.28, 128.05, 125.65, 125.41, 122.67, 120.92, 119.51, 101.75, 20.75, 14.05. ESI-MS *m*/*z*: 375.15 [M + H]^+^.

*1-(4-((1H-Pyrazolo[3,4-d]pyrimidin-4-yl)oxy)phenyl)-3-(2-chloro-5-(trifluoromethyl)phenyl)urea* (**1i**). Compound **1i** was prepared using the same procedure as described for the synthesis of **1a**. Yield: 82.3%; m.p. 239.2–240.3 °C; ^1^H-NMR (400 MHz, DMSO-*d*_6_) δ 14.13 (s, 1H), 9.70 (s, 1H), 8.66 (d, *J =* 3.2 Hz, 2H), 8.52 (s, 1H), 8.05 (s, 1H), 7.73 (d, *J =* 8.4 Hz, 1H), 7.60 (d, *J =* 8.8 Hz, 2H), 7.39 (d, *J =* 8.4 Hz, 1H), 7.29 (d, *J =* 8.8 Hz, 2H). ^13^C-NMR (100 MHz, DMSO-*d*_6_) δ 163.29, 157.14, 155.68, 152.91, 147.56, 137.32, 137.25, 132.22, 130.54, 128.80, 128.48, 125.49, 123.25, 120.47, 119.63, 117.67, 117.24, 102.01. ESI-MS *m*/*z*: 449.07 [M + H]^+^.

*1-(4-((1H-Pyrazolo[3,4-d]pyrimidin-4-yl)oxy)phenyl)-3-(3-fluoro-5-(trifluoromethyl)phenyl)urea* (**1j**). Compound **1j** was prepared using the same procedure as described for the synthesis of **1a**. Yield: 88.0%; m.p. 249.2–250.6 °C; ^1^H-NMR (400 MHz, DMSO-*d*_6_) δ 14.13 (s, 1H), 9.30 (s, 1H), 9.04 (s, 1H), 8.51 (s, 1H), 8.04 (s, 1H), 7.73 (s, 1H), 7.64 (d, *J =* 11.3 Hz, 1H), 7.58 (d, *J =* 8.6 Hz, 2H), 7.27 (d, *J =* 8.6 Hz, 2H), 7.22 (d, *J =* 8.2 Hz, 1H). ^13^C-NMR (100 MHz, DMSO-*d*_6_) δ 163.87, 163.58, 161.39, 157.11, 155.33, 152.81, 147.27, 143.05, 137.41, 132.20, 122.69, 120.40, 110.95, 108.89, 105.75, 105.54, 101.76. ESI-MS *m*/*z*: 433.10 [M + H]^+^.

*1-(4-((1H-Pyrazolo[3,4-d]pyrimidin-4-yl)oxy)phenyl)-3-(3-fluoro-5-methylphenyl)urea* (**1k**). Compound **1k** was prepared using the same procedure as described for the synthesis of **1a**. Yield: 81.6%; m.p. 242.8–245.2 °C; ^1^H-NMR (400 MHz, DMSO-*d*_6_) δ 9.06 (s, 1H), 8.45 (s, 1H), 8.28 (s, 1H), 7.33 (dd, *J* = 6.8, 2.4 Hz, 1H), 7.25–18 (m, 3H), 7.02 (t, *J* = 9.2 Hz, 1H), 6.69 (d, *J* = 8.8 Hz, 2H). ^13^C-NMR (100 MHz, DMSO-*d*_6_) δ 163.71, 157.48, 155.37, 155.13, 153.21, 146.68, 138.21, 136.15, 132.24, 124.65, 124.48, 122.62, 121.44, 119.57, 117.63, 115.40, 115.17, 101.73, 14.77. ESI-MS *m*/*z*: 379.12 [M + H]^+^.

*1-(4-((1H-Pyrazolo[3,4-d]pyrimidin-4-yl)oxy)phenyl)-3-(3-ethylphenyl)urea* (**1l**). Compound **1l** was prepared using the same procedure as described for the synthesis of **1a**. Yield: 61.2%; m.p. 219.5–221.5 °C; ^1^H-NMR (400 MHz, DMSO-*d*_6_) δ 14.13 (s, 1H), 8.78 (s, 1H), 8.65 (s, 1H), 8.52 (s, 1H), 8.01 (s, 1H), 7.57 (d, *J =* 8.7 Hz, 2H), 7.34 (s, 1H), 7.24–7.20 (m, 4H), 6.83 (d, *J =* 7.3 Hz, 1H), 2.58 (d, *J =* 7.2 Hz, 2H), 1.24–1.17 (m, 3H). ^13^C-NMR (100 MHz, DMSO-*d*_6_) δ 157.18, 155.39, 153.05, 146.75, 144.75, 140.08, 138.15, 129.09, 129.02, 122.66, 121.80, 120.74, 119.71, 117.99, 117.74, 116.09, 115.58, 101.76, 28.71, 15.96. ESI-MS *m*/*z*: 375.15 [M + H]^+^.

*1-(4-((1H-Pyrazolo[3,4-d]pyrimidin-4-yl)oxy)phenyl)-3-(3-nitrophenyl)urea* (**1m**). Compound **1m** was prepared using the same procedure as described for the synthesis of **1a**. Yield: 70.3%; m.p. 229.2–231.4 °C; ^1^H-NMR (400 MHz, DMSO-*d*_6_) δ 14.13 (s, 1H), 9.27 (s, 1H), 8.97 (s, 1H), 8.58 (s, 1H), 8.51 (s, 1H), 8.04 (s, 1H), 7.83 (d, *J =* 8.0 Hz, 1H), 7.74 (d, *J =* 8.1 Hz, 1H), 7.60 (d, *J =* 8.9 Hz, 2H), 7.57 (t, *J =* 8.3 Hz, 1H), 7.28 (d, *J =* 8.9 Hz, 2H). ^13^C-NMR (100 MHz, DMSO-*d*_6_) δ 155.35, 152.91, 148.52, 147.11, 141.43, 137.55, 130.46, 124.74, 122.73, 120.23, 116.71, 112.55, 101.74, 40.50, 40.29, 40.08, 39.87, 39.67, 39.46, 39.25. ESI-MS *m*/*z*: 392.10 [M + H]^+^.

*1-(4-((1H-Pyrazolo[3,4-d]pyrimidin-4-yl)oxy)phenyl)-3-(2-fluoro-5-(trifluoromethyl)phenyl)urea* (**1n**). Compound **1n** was prepared using the same procedure as described for the synthesis of **1a**. Yield: 78.6%; m.p. 178.4–179.0 °C; ^1^H-NMR (400 MHz, DMSO-*d*_6_) δ 14.13 (s, 1H), 9.70 (s, 1H), 8.66 (d, *J* = 3.2 Hz, 2H), 8.52 (s, 1H), 8.05 (s, 1H), 7.73 (d, *J* = 8.4 Hz, 1H), 7.60 (d, *J* = 8.4 Hz, 2H), 7.39 (d, *J* = 8.4 Hz, 1H), 7.29 (d, *J* = 8.8 Hz, 2H). ^13^C-NMR (100 MHz, DMSO-*d*_6_) δ 155.34, 153.38, 152.64, 152.56, 152.44, 147.24, 137.30, 130.76, 129.47, 125.66, 122.82, 120.88, 119.96, 119.19, 116.72, 116.42, 116.21, 115.69, 101.76. ESI-MS *m*/*z*: 406.12 [M + H]^+^.

*1-(4-((1H-Pyrazolo[3,4-d]pyrimidin-4-yl)oxy)phenyl)-3-(3-(methylthio)phenyl)urea* (**1o**). Compound **1o** was prepared using the same procedure as described for the synthesis of **1a**. Yield: 99.6%; m.p. 227.3–228.4 °C; ^1^H-NMR (400 MHz, DMSO-*d*_6_) δ 14.13 (s, 1H), 8.80 (d, *J* = 23.2 Hz, 2H), 8.51 (s, 1H), 8.02 (s, 1H), 7.56 (d, *J* = 8.8 Hz, 2H), 7.49 (s, 1H), 7.26–7.21 (m, 3H), 7.17 (d, *J* = 8.0 Hz, 1H), 6.88 (d, *J* = 7.6 Hz, 1H), 2.51 (s, 3H). ^13^C-NMR (100 MHz, DMSO-*d*_6_) δ 163.64, 157.12, 155.36, 152.95, 146.89, 140.61, 139.00, 137.92, 132.24, 129.66, 122.65, 119.92, 119.70, 115.68, 115.26, 101.75, 15.04. ESI-MS *m*/*z*: 393.11 [M + H]^+^.

*1-(4-((1H-Pyrazolo[3,4-d]pyrimidin-4-yl)oxy)phenyl)-3-(3-cyanophenyl)urea* (**1p**). Compound **1p** was prepared using the same procedure as described for the synthesis of **1a**. Yield: 86.7%; m.p. 235.2–237.4 °C; ^1^H-NMR (400 MHz, DMSO-*d*_6_) δ 14.15 (s, 1H), 9.05 (d, *J* = 39.2 Hz, 2H), 8.51 (s, 1H), 8.03 (d, *J* = 20.4 Hz, 2H), 7.70 (s, 1H), 7.57–7.44 (m, 4H), 7.27 (s, 2H). ^13^C-NMR (100 MHz, DMSO-*d*_6_) δ 163.60, 157.08, 155.36, 152.89, 147.05, 141.01, 137.61, 132.25, 130.59, 125.74, 123.32, 122.74, 121.19, 120.11, 111.99, 101.74. ESI-MS *m*/*z*: 372.11 [M + H]^+^.

*Methyl-3-(3-(4-((1H-pyrazolo[3,4-d]pyrimidin-4-yl)oxy)phenyl)ureido)benzoate* (**1q**). Compound **1q** was prepared using the same procedure as described for the synthesis of **1a**. Yield: 66.0%; m.p. 217.3–220.1 °C; ^1^H-NMR (400 MHz, DMSO-*d*_6_) δ 14.15 (s, 1H), 9.30 (s, 1H), 9.01 (s, 1H), 8.59 (s, 1H), 8.51 (s, 1H), 8.06 (s, 1H), 7.83 (d, *J* = 8.0 Hz, 1H), 7.74 (d, *J* = 8.0 Hz, 1H), 7.59 (d, *J* = 8.4 Hz, 3H), 7.28 (d, *J* = 7.6 Hz, 2H), 3.39 (s, 3H). ^13^C-NMR (100 MHz, DMSO-*d*_6_) δ 166.61, 163.63, 157.09, 155.37, 152.98, 146.92, 140.53, 137.83, 132.26, 130.56, 129.61, 123.19, 122.91, 122.69, 120.01, 119.02, 101.74, 52.58. ESI-MS *m*/*z*: 405.12 [M + H]^+^.

*1-(4-((1H-Pyrazolo[3,4-d]pyrimidin-4-yl)oxy)phenyl)-3-(5-methyl-2-nitrophenyl)urea* (**1r**). Compound **1r** was prepared using the same procedure as described for the synthesis of **1a**. Yield: 54.2%; m.p. 359.7–360.2 °C; ^1^H-NMR (400 MHz, DMSO-*d*_6_) δ 14.12 (s, 1H), 9.53 (s, 1H), 8.50 (d, *J* = 6.8 Hz, 1H), 8.26 (d, *J* = 6.0 Hz, 1H), 8.03 (s, 2H), 7.57 (s, 2H), 7.33–7.26 (m, 3H), 6.85 (s, 1H), 2.28 (d, *J* = 6.0 Hz, 3H). ^13^C-NMR (100 MHz, DMSO-*d*_6_) δ 163.61, 157.11, 155.36, 152.58, 146.98, 137.80, 137.45, 135.93, 132.24, 129.18, 124.42, 122.76, 122.18, 119.74, 119.42, 101.75, 21.32. ESI-MS *m*/*z*: 406.12 [M + H]^+^.

*1-(4-((1H-Pyrazolo[3,4-d]pyrimidin-4-yl)oxy)phenyl)-3-(3-bromophenyl)urea* (**1s**). Compound **1s** was prepared using the same procedure as described for the synthesis of **1a**. Yield: 85.5%; m.p. 234.5–237.0 °C; ^1^H-NMR (400 MHz, DMSO-*d*_6_) δ 14.16 (s, 1H), 8.95 (s, 1H), 8.91 (s, 1H), 8.51 (s, 1H), 8.06 (s, 1H), 7.88 (s, 1H), 7.56 (d, *J* = 7.2 Hz, 2H), 7.34 (d, *J* = 8.0 Hz, 1H), 7.26 (d, *J* = 6.8 Hz, 3H), 7.16 (d, *J* = 7.6 Hz, 1H). ^13^C-NMR (100 MHz, DMSO-*d*_6_) δ 155.38, 152.84, 146.95, 141.79, 137.73, 131.13, 124.78, 122.72, 122.13, 120.82, 120.02, 117.47, 101.74. ESI-MS *m*/*z*: 425.03 [M + H]^+^.

*1-(4-((1H-Pyrazolo[3,4-d]pyrimidin-4-yl)oxy)phenyl)-3-(3,4-dimethylphenyl)urea* (**1t**). Compound **1t** was prepared using the same procedure as described for the synthesis of **1a**. Yield: 87.7%; m.p. 249.9–250.6 °C; ^1^H-NMR (400 MHz, DMSO-*d*_6_) δ 14.12 (s, 1H), 8.75 (s, 1H), 8.52 (d, *J* = 5.6 Hz, 2H), 8.00 (s, 1H), 7.55 (d, *J* = 8.8 Hz, 2H), 7.24 (d, *J* = 8.8 Hz, 3H), 7.19 (d, *J* = 8.0 Hz, 1H), 7.04 (d, *J* = 8.0 Hz, 1H), 2.20 (s, 3H), 2.16 (s, 3H). ^13^C-NMR (101 MHz, DMSO-*d*_6_) δ 163.67, 157.11, 155.37, 153.02, 146.69, 138.19, 137.69, 136.73, 132.25, 130.04, 129.93, 122.62, 120.02, 119.67, 116.25, 101.74, 20.05, 19.07. ESI-MS *m*/*z*: 375.15 [M + H]^+^.

*4-Chloro-1-methyl-1H-pyrazolo[3,4-d]pyrimidine* (**6**). A suspension of compound **2** (1.88 g, 0.012 mol) and NaH (0.864 g, 0.036 mol) in dry DMF (10 mL) was stirred at 0 °C for 30 min. Iodomethane (2.56 g, 0.018 mol) was added, and the resulting mixture was stirred overnight. The reaction mixture was treated with water (100 mL) and extracted with EtOAc (60 mL × 3). The combined organic layers were removed in vacuo to give compound **6**. Yield: 70.8%; ^1^H-NMR (400 MHz, DMSO-*d*_6_) δ 8.85 (s, 1H), 8.44 (s, 1H), 4.08 (s, 3H).

*4-(1-Methyl-1H-pyrazolo[3,4-d]pyrimidin-4-yl)oxy)aniline* (**7**). To the mixture of *para*-aminophenol (0.28 g) in DMF (2 mL), Cs_2_CO_3_ (1.2 eq.) was added under the protection of nitrogen. The reaction mixture was stirred at room temperature for 1.5–2 h, and then compound **6** was added slowly. After stirring overnight, the reaction mixture was diluted with EtOAc (50 mL) and was washed with 1 M NaOH (60 mL × 1), water (80 mL × 1), 5% LiCl (50 mL × 2), and brine. The organic layer was dried over MgSO_4_ and concentrated to give compound **7**. Yield: 69.5%; ^1^H-NMR (400 MHz, DMSO-*d*_6_) δ 8.5 (s, 1H), 7.66 (s, 1H), 6.97 (d, *J =* 8.8 Hz, 2H), 6.67 (d, *J =* 8.8 Hz, 2H), 5.37 (s, 2H), 4.01 (s, 3H).

*1-(3-Chlorophenyl)-3-(4-((1-methyl-1H-pyrazolo[3,4-d]pyrimidin-4-yl)oxy)phenyl)urea* (**1u**). Compound **1u** was prepared from compound **7** and 3-chlorophenyl isocyanate using the same procedure as described for the synthesis of **1a**. Yield: 83.4%; m.p. 309.8–312.0 °C; ^1^H-NMR (400 MHz, DMSO-*d*_6_) δ 8.94 (s, 1H), 8.89 (s, 1H), 8.55 (s, 1H), 8.04 (s, 1H), 7.73 (s, 1H), 7.57 (d, *J =* 8.0 Hz, 2H), 7.30–7.25 (m, 4H), 7.03 (s, 1H), 4.05 (s, 3H). ^13^C-NMR (100 MHz, DMSO-*d*_6_) δ 163.70, 155.39, 155.27, 152.86, 146.96, 141.65, 137.81, 133.61, 131.32, 130.80, 122.65, 121.90, 120.04, 118.02, 117.10, 102.19. ESI-MS *m*/*z*: 395.09 [M + H]^+^.

*1-(4-Chloro-3-(trifluoromethyl)phenyl)-3-(4-((1-methyl-1H-pyrazolo[3,4-d]pyrimidin-4-yl)oxy)phenyl)urea* (**1v**). Compound **1v** was prepared using the same procedure as described for the synthesis of **1u** by replacing 3-chlorophenyl isocyanate with 4-chloro-3-(trifluoromethyl)phenyl isocyanate. Yield: 89.3%; m.p. 212.1–213.0 °C; ^1^H-NMR (600 MHz, DMSO-*d*_6_) δ 9.21 (s, 1H), 8.98 (s, 1H), 8.54 (s, 1H), 8.12 (s, 1H), 8.04 (s, 1H), 7.62-7.68 (m, 2H), 758 (d,*J =* 9.0 Hz, 2H), 7.26 (d,*J =* 9.0 Hz, 2H), 4.04 (s, 3H). ^13^C-NMR (100 MHz, DMSO-*d*_6_) δ 163.65, 155.35, 155.24, 152.88, 147.10, 139.73, 137.57, 132.37, 131.29, 127.26, 126.95, 123.47, 122.63, 120.29, 117.17, 102.17, 34.43. ESI-MS *m*/*z*: 461.81 [M − H]^−^.

### 3.2. Kinase Inhibitory Activity

The BRAF^V600E^ inhibitory activity was evaluated by using the Z’-LYTE technology platform (Life Technologies, Carlsbad, CA, USA), and Sorafenib was employed as the positive control. The experiments were performed according to the instructions of the manufacturer, and single point concentration testing with two independent data points (*n* = 2). According to the anti-proliferative activities and percent inhibition values of compounds, we chose compounds **1l**, **1u** and **1v** to evaluate kinase activity at ten concentration gradients from 1000 to 0.0508 nM (3-fold dilution). The IC_50_ values were calculated from the inhibition curves from two separate experiments.

### 3.3. Cell proliferation Inhibition Assay

The anti-proliferative activities of compounds were evaluated against A375, HT-29, PC-3, A549 and MDCK cell lines by the standard MTT assay in vitro, with Sorafenib as the positive control. All cell lines were purchased from Cell Bank of China Science Academy (Shanghai, China). All chemicals and solvents were purchased from Sigma-Aldrich (St. Louis, MO, USA) or Gibco (Gibco BRL, Grand Island, NY, USA). The PC-3 and A375 cell lines was maintained in Dulbecco's modified eagle (DEME) medium supplement with 10% fetal bovine serum (FBS) and 1% penicillin–streptomycin. The others were cultured in Roswell Park Memorial Institute (RPMI) 1640 medium supplement with 10% fetal bovine serum (FBS) and 1% penicillin–streptomycin. Approximately 5 × 10^3^ cells, suspended in medium, were plated into each well of a 96-well plate and grown at 37 °C in a humidified atmosphere with 5% CO_2_ for 24 h. The following day, various concentrations of tested compounds were added to the culture medium and incubated for 48 h. Fresh MTT was added to each well at the terminal concentration of 5 mg/mL in phosphate buffered saline (PBS), and incubated with cells at 37 °C for 4 h. The formazan crystals in each well were dissolved in 150 μL DMSO, and the absorbency at 570 nm was measured with an enzyme-linked immunosorbent assay plate reader. All of the compounds were tested three times in each of the cell lines.

### 3.4. Flow-Activating Cell Sorting Analysis

The effect of compound **1v** on cell cycle phase distribution of human malignant melanoma A375 was assessed using flow cytometry. When the cells grew to about 70% confluence in 6-well microplates, they were treated with compound **1v** at given concentrations (6.11 μmol/L, 12.21 μmol/L, 24.42 μmol/L). After 48 h, cells were harvested by trypsinization, washed with PBS, and fixed in 70% ice cold (4 °C) ethanol overnight. They were then washed with PBS, incubated with Ribonuclease (RNase) (50 mg/mL final concentration) at 37 °C for 30 min, stained with propidium iodide (50 mg/mL final concentration), and analyzed by flow cytometry (BD FACS Canto II, BD Biosciences, San Jose, CA, USA).

### 3.5. Western Blot Analysis

Total cells were harvested after treatment, washed twice with cold PBS, and lysed with RIPA lysis buffer (KeyGEN BioTECH, Nanjing, China) including 1% cocktail (Selleck Chemicals, Houston, TX, USA). Lysates were centrifuged at 12,000× *g* at 4 °C for 15 min, and the supernatant was collected. Total protein concentration was quantified using the bicinchoninic acid (BCA; Beyotime Institute of Biotechnology, Nanjing, China) protein assay kit (Sigma Chemical Co., St. Louis, MO, USA). The proteins were separated electrophoretically using 10% sodium dodecyl sulfate polyacrylamide gel electrophoresis (SDS-PAGE) and then the gel was transferred onto polyvinylidene fluoride (PVDF; Millipore, Billerica, MA, USA) membranes. The membranes were subsequently blocked with the use of 5% milk in tris-buffered saline Tween (TBST) for 1 h and incubated overnight with antibodies against p217/p221-MEK (Pmek, Cell Signaling Technology, Beverly, MA, USA), Glyceraldehyde-3-phosphate dehydrogenase (GAPDH, Santa Cruz Biotechnology, Inc., Santa cruz, CA, USA) in TBST containing 5% defatted milk at 4 °C followed by incubation with horseradish peroxidase-linked secondary antibodies (Beyotime Institute of Biotechnology, Nanjing, China) at 4 °C for additional 1 h. The immunobands were detected with an enhanced chemiluminescence kit (Beyotime Institute of Biotechnology, Nanjing, China).

### 3.6. Molecular Modelling

Molecular docking was performed with Surflex–Dock program that is interfaced with Sybyl 7.3 [27]. The crystal structure of BRAF^V600E^ kinase in complex with Sorafenib (PDB ID: 1UWJ [28]) was obtained from the PDB. All the water and ligands were removed and the random hydrogen atoms were added. The structures of the synthesized compounds were generated and minimized using tripos force fields. All the other default parameters were used. The highest-scored conformation based on the Surflex–Dock scoring functions, was selected as the final bioactive conformation.

The MD simulations were performed using AMBER 12 software package (San Francisco, CA, USA) [29,30] according to our previously published protocol [31]. The missing residues, which were not solved in the crystal structures, were modeled using the loop building routine in Modeler [32]. Geometry optimization and the electrostatic potential calculations for **1a** and **1v** were carried out at the HF/6-31G* level of the Gaussian03 suite [33]. The atomic partial charge was obtained by using the restrained electrostatic potential (RESP) fitting technique [34] implemented in AmberTools [35]. The force field parameters for **1a** and **1v** were generated by the general amber force field (GAFF) [36] using the Antechamber program. The AMBER 99SB force field [37] was used to simulate the protein structure and the ionization states of amino acid residues were set according to the standard protocol. The model was neutralized by adding suitable counterions and were solvated in a truncated octahedron box of the transferable interaction potential (TIP3P) [38] water molecules with a margin distance of 12 Å. The fully solvated and neutralized system was subjected to energy minimization. Following minimization, the system was gradually heated from 0 to 300 K in 50 ps using a Langevin thermostat with a coupling coefficient of 1.0/ps with a force constant 2.0 kcal·mol^−1^·Å^−2^ on the complex. Then, 50 ps of density equilibration with a force constant 2.0 kcal·mol^−1^·Å^−2^ on the complex was performed. Subsequently the systems were again equilibrated for 500 ps by releasing all the restraints. Finally, the 50-ns MD production was performed at 300 K with 1.0 atm pressure. During the MD simulations, the long-range Coulombic interactions were handled using the particle mesh Ewald (PME) method [39]. The cutoff distance for the long-range vdW energy term was set at 10.0 Å. Periodic boundary conditions were applied to avoid edge effects in all calculations. The SHAKE algorithm [40] was employed on all atoms covalently bond to hydrogen atoms, allowing for an integration time step of 2 fs. Coordinate trajectories were recorded every 10 ps throughout all equilibration and production runs. The binding free energies were calculated by using the MM-PBSA and MM-GBSA [41] procedure in AMBER12. An average of 1000 snapshots were extracted from the last 10 ns MD trajectory for the calculations. The calculations of entropic terms are time-consuming and have low prediction accuracy, thus the conformational entropy change was omitted.

## 4. Conclusions

We designed and synthesized pan-RAF bisarylurea inhibitors bearing 1*H*-pyrazolo[3,4-*d*]pyrimidine scaffold targeting DFG-out conformation on the basis of structure-based drug design using docking of compound **1a** into the active site of X-ray cocrystal structures of BRAF. Molecular docking indicated that these compounds bind to the ATP-binding site of DFG-out BRAF conformation, with the urea portion forming hydrogen bonds with Asp594 and Glu501, and the terminal-substituted phenyl projecting into the large hydrophobic pocket. By optimizing the substituents at both terminal phenyl ring and 1-NH, a series of compounds were obtained. In particular, the most promising compound, **1v**, exhibited potent inhibitory activity against not only BRAF^V600E^ (IC_50_ = 23.6 nM) but also wild-type BRAF (IC_50_ = 51.5 nM) and C-RAF (IC_50_ = 8.5 nM), and effective cellular anti-proliferative potencies against A375, HT-29, PC-3 and A549 cell lines, as well as a very good selectivity profile. Furthermore, flow-activated cell sorting analysis revealed that compound **1v** arrested the cell cycle of the A375 cell line in the G0/G1 phase with a concentration-dependent effect. Moreover, compound **1v** showed significant suppression of MEK phosphorylation in A375 and HT-29 cell lines. Taken together, the optimal compound **1v** showed excellent in vitro potency as a pan-RAF inhibitor. In addition, molecular dynamics simulation and binding free energy calculations were conducted to analysis the interaction of synthesized compounds with BRAF, and the results suggested that ligand **1v** showed improved potency compared to that of **1a**.

## Figures and Tables

**Table 1:**
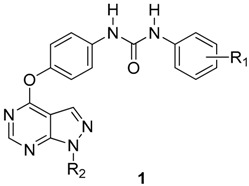
The kinase inhibitory activities of synthesized compounds.

Compound	Structure		BRAF^V600E^ (%) ^a^	
	R_1_	R_2_	1000 nM	100 nM
**1a**	3-CF_3_-4-Cl	H	90	37
**1b**	3-Me	H	46	N.T ^b^
**1c**	3,4-di-Cl	H	83	27
**1d**	4-Cl	H	51	N.T
**1e**	H	H	1	N.T
**1f**	2-Cl-5-Me	H	37	N.T
**1g**	3-Cl	H	54	N.T
**1h**	2,3-di-Me	H	27	N.T
**1i**	2-Cl-5-CF_3_	H	83	52
**1j**	3-F-5-CF_3_	H	67	37
**1k**	3-Me-4-F	H	42	N.T
**1l**	3-Et	H	69	60
**1m**	3-NO_2_	H	62	3
**1n**	2-F-5-CF_3_	H	87	30
**1o**	3-SMe	H	69	29
**1p**	3-CN	H	48	N.T
**1q**	3-CO_2_Me	H	32	N.T
**1r**	2-NO_2_-5-Me	H	22	N.T
**1s**	3-Br	H	71	10
**1t**	3,4-di-Me	H	89	28
**1u**	3-Cl	Me	93	61
**1v**	3-CF_3_-4-Cl	Me	97	81
**Sorafenib**			94	75

^a^ Values are the average of two independent experiments; ^b^ N.T: Not Tested.

**Table 2:** The kinase inhibitory activities of selected compounds.

Compound	Structure		Kinase Tested (IC_50_, nM) ^a^
	R_1_	R_2_	BRAF^V600E^
**1l**	3-Et	H	51.3
**1u**	3-Cl	Me	38.1
**1v**	3-CF_3_-4-Cl	Me	23.6
**Sorafenib**			52.3

^a^ Values are the average of two independent experiments. IC_50_: half maximal inhibitory concentration.

**Table 3:** The in vitro anti-proliferative activity (IC_50_, μM) ^a^.

Compound	R_1_	R_2_	A375	HT-29	PC-3	A549	MDCK
**1a**	3-CF_3_-4-Cl	H	29.28	>80	19.53	19.75	17.76
**1b**	3-Me	H	>80	>80	>80	75.60	>80
**1c**	3,4-di-Cl	H	29.70	15.17	11.16	16.18	35.47
**1d**	4-Cl	H	78.82	>80	18.35	>80	>80
**1e**	H	H	>80	>80	>80	>80	>80
**1f**	2-Cl-5-Me	H	62.27	61.71	1.12	5.24	>80
**1g**	3-Cl	H	>80	>80	>80	70.12	>80
**1h**	2,3-di-Me	H	>80	>80	11.72	>80	>80
**1i**	2-Cl-5-CF_3_	H	26.95	62.41	1.12	60.83	>80
**1j**	3-F-5-CF_3_	H	30.45	50.66	45.98	60.32	>80
**1k**	3-Me-4-F	H	>80	>80	22.71	62.10	>80
**1l**	3-Et	H	75.57	71.25	>80	19.93	>80
**1m**	3-NO_2_	H	64.04	31.68	23.42	>80	>80
**1n**	2-F-5-CF_3_	H	5.39	27.82	40.28	32.09	23.12
**1o**	3-SMe	H	>80	>80	5.34	29.97	>80
**1p**	3-CN	H	61.78	39.39	30.91	59.48	>80
**1q**	3-CO_2_Me	H	>80	>80	22.54	>80	>80
**1r**	2-NO_2_-5-Me	H	>80	>80	>80	>80	>80
**1s**	3-Br	H	65.44	>80	5.52	>80	>80
**1t**	3,4-di-Me	H	75.23	>80	>80	>80	>80
**1u**	3-Cl	Me	13.20	>80	68.85	>80	>80
**1v**	3-CF_3_-4-Cl	Me	12.21	14.72	23.41	12.49	>80
**Sorafenib**			6.81	8.12	13.79	13.72	14.67

^a^ IC_50_ values are presented as mean values of three independent experiments done in quadruplicates. Coefficients of variation were < 10%. MDCK: Madin-Darby canine kidney.

**Table 4:** Kinase selectivity of compound **1v**.

Kinase Tested	Inhibition % at 1 μΜ	Kinase Tested	Inhibition % at 1 μΜ
BRAF (wt)	105 (51.5) ^a^	EPHA2	2
RAF1 (CRAF)	102 (8.5) ^a^	FLT4 (VEGFR3)	58
ABL2 (Arg)	4	ABL1	5
EGFR (ErbB1)	8	ALK	7
ErbB2 (HER2)	−7	AURKA (Aurora A)	17
ErbB4 (HER4)	7	AURKB (Aurora B)	42
PTK2 (FAK)	13	LCK	20
FLT1 (VEGFR1)	31	LTK (TYK1)	3
MET (c-Met)	6	MST1R (RON)	−2
VEGFR-2	56		

^a^ IC_50_ values are presented as mean values of two independent experiments done in quadruplicates. Coefficients of variation were < 10%. RAF: Ras activating factor; ABL: Abl family nonreceptor tyrosine kinase; EGFR: epidermal growth factor receptor; HER: human epidermal growth factor receptor; FAK: focal adhesion kinase; FLT: fms-like tyrosine kinase; MET: mesenchymal-epithelial transition; VEGFR: vascular endothelial growth factor receptor; ALK: anaplastic lymphoma kinase; AURK: Aurora kinase; LCK: lymphocyte kinase; MST1R: macrophage-stimulating 1 receptor.

**Table 5:** Effect of compound **1v** on cell cycle distribution in the A375 cell line.

Concentration	Sub-G1 (%)	G0/G1 (%)	S (%)	G2/M (%)
0	1.25	39.43	38.68	19.09
6.11 μmol/L	4.39	42.53	38.25	10.94
12.21 μmol/L	2.12	47.94	38.13	7.39
24.42 μmol/L	5.48	52.73	36.19	5.48

**Table 6:** Main hydrogen-bond interactions between inhibitors and BRAF^V600E^.

Donor	Acceptor	1a			1v		
		Dist ^a^ (Å)	Ang ^b^ (°)	Occ ^c^ (%)	Dist (Å)	Ang (°)	Occ (%)
Lig@N2H	Glu 501@OE2	2.854	23.46	99.60	2.845	23.56	100.00
Lig@N3H	Glu 501@OE2	2.897	31.59	99.50	2.896	32.71	98.90
Asp 594@NH	Lig@O	2.970	25.99	98.20	3.005	27.22	96.80
Cys 532@NH	Lig@N	3.117	31.45	83.40	3.209	29.20	68.50
Lig@N1H	Cys 532@O	3.015	25.28	53.00	-	-	-

^a^ The average distance with standard error (SE = standard deviation/N^1/2^) in parentheses between hydrogen-acceptor atom and hydrogen-donor atom in the investigated time period; ^b^ The average angle with standard error in parentheses for hydrogen bonds in the investigated time period; ^c^ Occupancy is in unit of percentage of the investigated time period.

**Table 7:** Binding free energy values for BRAF^V600E^ complexes obtained by MM-PBSA and MM-GBSA (kcal/mol) ^a^.

	1a	1v
ΔE_vdW_	−56.67 (0.09)	−59.06 (0.10)
ΔE_ele_	−41.49 (0.15)	−39.50 (0.12)
ΔE_MM_	−98.16 (0.14)	−98.56 (0.12)
ΔG_ele,sol_ (GB)	48.50 (0.07)	46.63 (0.07)
ΔG_np,sol_ (GB)	−7.42 (0.01)	−7.64 (0.01)
ΔG_sol_ (GB)	41.08 (0.07)	38.99 (0.07)
ΔG_ele,sol_ (PB)	54.88 (0.08)	55.03 (0.10)
ΔG_np,sol_ (PB)	−4.14 (0.00)	−4.49 (0.00)
ΔG_sol_ (PB)	50.74 (0.08)	50.53 (0.09)
ΔG_pred_ (GB)	−57.08 (0.12)	−59.57 (0.11)
ΔG_pred_ (PB)	−47.42 (0.12)	−48.03 (0.12)

^a^ ΔG_pred_: the calculated binding free energy by MM-PBSA and MM-GBSA method, ΔG_pred_ = ΔG_ele_ + ΔG_vdW_ + ΔG_np,sol_ + ΔG_ele,sol_.
